# Specific biomarkers for stochastic division patterns and starvation-induced quiescence under limited glucose levels in fission yeast

**DOI:** 10.1111/j.1742-4658.2011.08050.x

**Published:** 2011-04

**Authors:** Tomáš Pluskal, Takeshi Hayashi, Shigeaki Saitoh, Asuka Fujisawa, Mitsuhiro Yanagida

**Affiliations:** 1 Okinawa Institute of Science and Technology Promotion CorporationOkinawa, Japan; 2 CREST Research Project, Japan Science and Technology Corporation (JST), Graduate School of Biostudies, Kyoto UniversityJapan; 3 Division of Cell Biology, Institute of Life Science, Kurume UniversityFukuoka, Japan

**Keywords:** CDP-choline, ergothioneine, glutathione, longevity, trehalose

## Abstract

Glucose as a source of energy is centrally important to our understanding of life. We investigated the cell division–quiescence behavior of the fission yeast *Schizosaccharomyces pombe* under a wide range of glucose concentrations (0–111 mm). The mode of *S. pombe* cell division under a microfluidic perfusion system was surprisingly normal under highly diluted glucose concentrations (5.6 mm, 1/20 of the standard medium, within human blood sugar levels). Division became stochastic, accompanied by a curious division-timing inheritance, in 2.2–4.4 mm glucose. A critical transition from division to quiescence occurred within a narrow range of concentrations (2.2–1.7 mm). Under starvation (1.1 mm) conditions, cells were mostly quiescent and only a small population of cells divided. Under fasting (0 mm) conditions, division was immediately arrested with a short chronological lifespan (16 h). When cells were first glucose starved prior to fasting, they possessed a substantially extended lifespan (∼14 days). We employed a quantitative metabolomic approach for *S. pombe* cell extracts, and identified specific metabolites (e.g. biotin, trehalose, ergothioneine, *S*-adenosyl methionine and CDP-choline), which increased or decreased at different glucose concentrations, whereas nucleotide triphosphates, such as ATP, maintained high concentrations even under starvation. Under starvation, the level of *S*-adenosyl methionine increased sharply, accompanied by an increase in methylated amino acids and nucleotides. Under fasting, cells rapidly lost antioxidant and energy compounds, such as glutathione and ATP, but, in fasting cells after starvation, these and other metabolites ensuring longevity remained abundant. Glucose-starved cells became resistant to 40 mm H_2_O_2_ as a result of the accumulation of antioxidant compounds.

## Introduction

Glucose is made by photosynthesis in plants and certain bacteria. It is the essential source of cellular energy for all organisms as its metabolism to CO_2_ and H_2_O generates ATP by glycolysis in the cytosol and subsequent respiratory electron transport coupled to oxidative phosphorylation in the mitochondria. Glucose forms polymerized complexes, such as starch, glycogen or cellulose, for storage and architecture. Glucose is circulated within the human body via the bloodstream for supply to body cells. Hormones, such as insulin, control the uptake, storage and consumption of glucose in human bodies [1]. The level of glucose in the human blood is tightly regulated as a part of metabolic homeostasis, fluctuating during the day and peaking after meals. Normally, the human blood glucose reference level (the daily lowest level before breakfast) is maintained within a range of approximately 3.9–6.1 mm [2]. Glucose levels rise briefly after meals for an hour or two. In diabetic patients, normal regulation of blood glucose levels is disrupted for various reasons, resulting in a generally prolonged high concentration of glucose in the blood [3].

The fission yeast *Schizosaccharomyces pombe* is an excellent model eukaryote [4–6] for a number of cell biologic issues, such as cell division cycle control [7], meiosis [8], actin- and microtubule-mediated cytoskeletal organization [9], centromere/kinetochore-based chromosome segregation [10], DNA damage repair [11], replication [12], transcription [13] and gene silencing [14]. *S. pombe* contains mitochondria with a small-sized DNA, similar to that in humans [15], lysosome-like vacuoles [16], peroxisomes [17], lipid droplets, endosomes and endoplasmic reticulum, all of which may be important for the support of cellular glucose metabolism. It has been proposed to utilize *S. pombe* as a model for cellular aging [18,19]. Glucose is reported to enhance aging in many organisms, including *S. pombe* [20]. Establishing *S. pombe* as a model for glucose metabolism would allow for the use of powerful genetic methods available for this organism. If cellular regulatory systems for glucose utilization are highly conserved between humans and fission yeast, *S. pombe* studies may be useful to understand human glucose-related diseases such as diabetes. Such studies must, however, be performed at a similar glucose concentration to that supplied to human cells via the bloodstream, as in excess glucose the phenotypes associated with diseases may not be observed. In general, the glucose concentration in standard laboratory culture media for fungi is approximately 20–30 times higher than that in normal human blood [21]. Even the standard Dulbecco’s medium (DMEM) for human cell lines contains several times higher glucose levels [22].

In this study, we evaluated the mode of *S. pombe* cell division under a wide range of glucose concentrations, using the perfusion system, and show that *S. pombe* cells can efficiently increase in number at glucose concentrations similar to those in normal human blood. Previously, we have reported the comprehensive analysis of the *S. pombe* metabolome using LC/MS, with the semi-quantitative analysis of more than 100 principal metabolites [23]. We applied such analysis for *S. pombe* cells cultured under a wide range of different glucose conditions. Some specific metabolites may be designated as biomarkers, because of their distinct diagnostic responses (increase or decrease) at different glucose concentrations.

## Results

### Multiplication of *S. pombe* at a glucose level equivalent to that in human blood

**Fig. 1:**
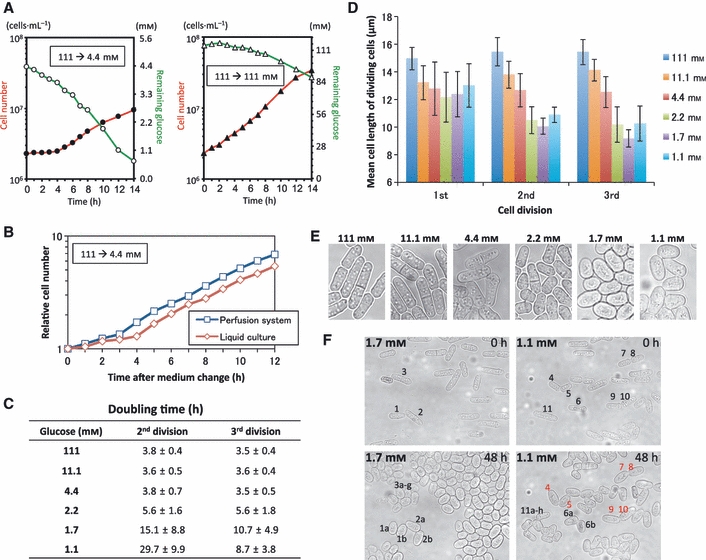
Cell behavior of *S. pombe* under limited glucose concentrations. (A) Cells cultured in standard medium containing 111 mm glucose were shifted to medium containing 4.4 mm glucose (left) or to control culture containing the same amount (111 mm) of glucose (right). The cell number increase and the level of glucose remaining in the liquid culture were measured at 26 °C for 14 h. (B) Comparison of the cell number increase between the two culture systems. Red: cells cultivated in a water bath shaker in liquid EMM2 culture (111 mm glucose), collected by vacuum filtration and switched to a new medium (4.4 mm glucose). Blue: cells fixed in a microscopic perfusion system, which constantly supplied fresh medium, switched from 111 to 4.4 mm glucose. (C) The doubling time (h) under different glucose concentrations was obtained by the observation of movies taken using the microscopic perfusion system (see text). (D) The mean cell length of dividing cells under the perfusion system was determined for different glucose concentrations. (E) Micrographs of cells cultured in different glucose concentrations. (F) Micrographs of cells in the same microscope field at 0 h (top) and 48 h (bottom) in culture medium containing 1.7 mm (left) and 1.1 mm (right) glucose. Cells identified by red numbers did not divide, whereas cells identified by black numbers performed one or several divisions.

The standard synthetic medium EMM2 for *S. pombe* contains 2% glucose (111 mm, 2000 mg·dL^−1^). It should be noted that glucose is the sole carbon source in EMM2 as the nitrogen source is NH_4_Cl (not amino acids). To examine whether *S. pombe* grows and divides under a glucose concentration similar to that in human blood, *S. pombe* was cultured in 20 mL of EMM2 medium containing 25-fold-diluted glucose (4.4 mm), the concentration equivalent to the normal level (80 mg·dL^−1^) in human blood before breakfast. As shown in Fig. 1A, the cell number increase (red line) and the remaining glucose concentration (green line) were measured at 26 °C in the culture transferred from 111 to 4.4 mm glucose at 0 h (left panel) and in the control culture transferred to the same 111 mm glucose medium (right panel; Fig. S1 obtained at 30 °C). After transfer to 4.4 mm glucose, the cell number increased only approximately five-fold from 2 × 10^6^ mL^−1^, and the remaining glucose was exhausted after approximately 10–14 h. In the control 111 mm glucose medium, however, the cell number continued to increase approximately 15-fold after 10–14 h, and the glucose concentration at that time remained high (85 mm).

Glucose was nearly exhausted at the end of the experiment for the initial 4.4 mm glucose, and the doubling times from the cell number increase during the earlier period (4–8 h, ∼3.0 mm remaining glucose) were 3.3 and 4.2 h at 30 and 26 °C, respectively. In the 111 mm glucose medium, the doubling times were 2.5 and 3.5 h at 30 and 26 °C (4–8 h, ∼100 mm remaining glucose), respectively. Considering the large difference (25-fold) in glucose concentrations, the difference in the doubling time was surprisingly small.

### Decreased cell size helps to maintain the doubling time under low glucose conditions

To avoid the problem of a decrease in glucose concentration when determining various parameters of cell division using static culture conditions (no exchange of the medium over time), we employed a low-volume specimen chamber that was continuously supplied with fresh culture medium (Onix™ Microfluidic Perfusion System, CellASIC, Hayward, CA, USA) at a flow rate of 3 μL·h^−1^. Using a DeltaVision microscope system (Applied Precision, Issaquah, WA, USA), which was installed in a room kept at a constant temperature (26 °C), movies were obtained of living cells that were initially cultured in medium containing 111 mm glucose and then changed to medium containing 111 (control), 11.1, 4.4, 2.2, 1.7, 1.1 or 0 mm glucose (Movies S1–S7). Cells divided frequently in 111, 11.1 and 4.4 mm glucose, but the division rate decreased in 2.2 mm, decreased further in 1.7 and 1.1 mm, and stopped completely in 0 mm glucose. The period of temporal cell division arrest observed after the culture change from 111 to 4.4 mm glucose was shorter in the perfusion system (blue, Fig. 1B) than in the liquid culture (red), perhaps as a result of the simplicity of the culture change manipulation: the microscopic perfusion was continuous and did not require a filter to collect cells for the intermittent medium change, which probably caused a physical shock to the cells. A number of cells in the movies were followed over time (24–48 h).

The doubling time was obtained for the second and third division (Fig. 1C), as the first division time showed large variations as a result of the diverse cell cycle points at the time of the glucose concentration shift. The mean values (3.5–3.8 h) for the doubling time of cells cultured in 111, 11.1 and 4.4 mm glucose were virtually identical, but increased (5.6 h) in 2.2 mm glucose. At lower glucose concentrations (1.7 and 1.1 mm), division was scarce with a long doubling time and large standard deviations.

We then examined how cells in 4.4 mm glucose managed to divide with a doubling time that matched that in 111 mm glucose. Cells became short and pear-shaped under glucose limitation. As shown in Fig. 1D, E, the cell length at the time of division was reduced from a mean of 15 μm in 111 mm glucose to approximately 13 μm in 4.4 mm and 10 μm in 2.2 mm glucose. In 11.1 mm glucose, the doubling time was identical and the cell length was intermediate between that at 111 and 4.4 mm glucose. Considering the reduced cell size and accumulation of certain stress-related metabolites (see below), we designated the 4.4 mm glucose concentration as the ‘diet’ condition. The 11.1 mm glucose concentration was designated as the ‘regular’ condition, as such effects were small.

### Glucose starvation causes semiquiescence

**Fig. 6:**
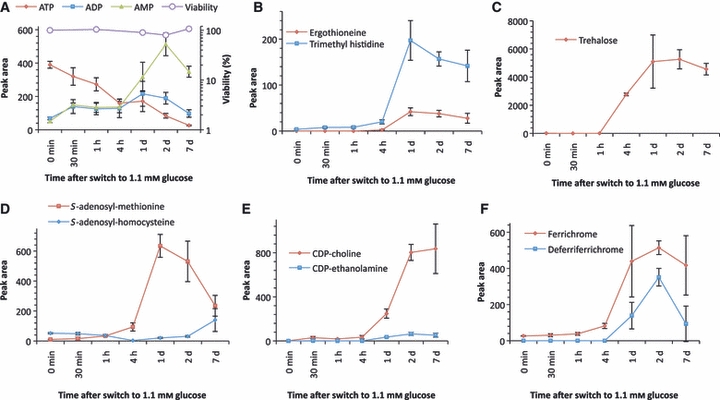
The time course change of the peak areas of key metabolites in cells switched to the starvation condition (1.1 mm glucose) from 111 mm glucose. *S. pombe* cells cultivated in mid-logarithmic phase (5 × 10^6^ cells·mL^−1^) in standard EMM2 medium containing 111 mm glucose were shifted to the starvation condition (1.1 mm glucose) and metabolites were extracted after 30 min, 1 h, 4 h, 1 day, 2 days and 7 days. Three samples were prepared at each time point. Mean peak areas with standard deviations of the following metabolites are shown: (A) ATP, ADP and AMP; cell viability is also shown in this plot; (B) ergothioneine, trimethyl-histidine; (C) trehalose; (D) *S*-adenosyl-methionine, *S*-adenosyl-homocysteine; (E) CDP-choline, CDP-ethanolamine; (F) ferrichrome, deferriferrichrome.

In 2.2 mm glucose, the doubling time increased considerably (5.6 h versus 3.5 h) and the cell length at the time of division decreased (10 μm versus 15 μm) in comparison with the 111, 11.1 and 4.4 mm glucose conditions. Further reduction of glucose to 1.7 and 1.1 mm induced semiquiescence among many cells when shifted from 111 mm glucose. In Fig. 1F, the top images were taken at the beginning of incubation and the bottom images were taken after 48 h in 1.7 mm (left) and 1.1 mm (right) glucose. The cells indicated by the black numbers divided one to four times during the 48 h, and those marked by the red numbers did not divide. The starving glucose conditions caused by these concentrations induced quiescence and infrequent division. The ability to divide seemed quite variable among individual cells; certain cells were either nondividing or divided up to four times. Cell viability, however, did not decrease at all during the 48 h, and remained close to 100% for 7 days (see Fig. 6A), a result consistent with a previously published observation that the chronological lifespan of *S. pombe* increases in a limited glucose environment [20].

### Under starvation, stochastic division and quiescence prevail

**Fig. 2:**
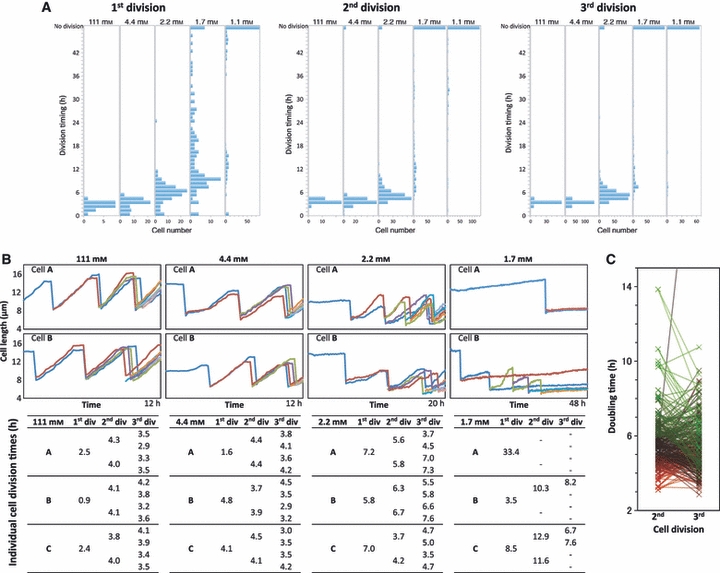
Cell division timing under restricted glucose. (A) The division timing (h) from the first to the third division was measured for a number of cells cultured in the perfusion system in media with different glucose concentrations (111, 4.4, 2.2, 1.7 and 1.1 mm). (B) Cell division timing was monitored by measuring the cell length vs. time (h) for three individual cells (A, B and C) cultured in 111, 4.4, 2.2 or 1.7 mm glucose. The top panels show detailed cell length measurements for two individual cells (A and B) vs. time. The bottom table shows the time span between divisions for cells A, B and C. (C) Inheritance of the doubling time for cells cultured in medium containing 2.2 mm glucose. The doubling time of the second division (left) was classified by three colors (short, red; medium, black; long, green) and connected to the doubling time of the third division (right) of the same cell.

We measured the doubling time (obtained from movies) for a number of individual cells in the perfusion system, and the distribution under different glucose concentrations is shown in Fig. 2A. In 1.7 and 1.1 mm glucose, the number of nondividing cells increased, and the doubling time became broadly distributed. In 2.2 mm glucose, most cells divided, although the doubling time was longer than that of cells cultured in 111 and 4.4 mm glucose.

Based on the narrow doubling time distribution in the second and third divisions, the doubling time was quite uniform for 111 and 4.4 mm glucose, and the stochastic nature of cell division became apparent in 2.2 mm glucose, and prominent in 1.7 and 1.1 mm glucose conditions. A sharp transition thus existed between the 2.2 and 1.7 mm glucose conditions: the second division doubling time was approximately 7 h for 2.2 mm glucose and 4–48 h for 1.7 mm glucose. Nondividing cells were scarce in 2.2 mm glucose, but plentiful in 1.7 and 1.1 mm glucose; hence, we designated 1.7 and 1.1 mm glucose conditions as ‘substarvation’ and ‘starvation’, respectively.

### Division timing is inherited from mother to daughters under starvation

We characterized more detailed division patterns by measuring the time course of changes in cell length by following a number of cell lineages. In 111 and 4.4 mm glucose, each of three examples of lineages indicated that the division patterns of mother–daughter–granddaughter cells were quite similar (Fig. 2B). A cell length plateau normally exists, which indicates that mitosis and cell separation are arrested with an increase in cell length. In 4.4 mm glucose, the initial cell division arrest seemed to occur at any stage of the cell cycle and lasted for approximately 4 h.

In the 2.2 and 1.7 mm glucose conditions, an irregular cell division mode was obvious for individual cell lineages (Fig. 2B, two right panels). The doubling time occasionally exceeded 7 h in 2.2 mm and 24 h in 1.7 mm glucose. It should be noted that certain lineages continuously divided, but others did not divide at all, during the observation period. In 2.2 mm glucose, the mother cells that showed a short doubling time tended to produce daughters that also showed a short doubling time. This was substantiated by evaluating the doubling time between the second and third division from the cell lineages in 2.2 mm glucose (Fig. 2C). Cells with a short doubling time (red lines) also had short intervals between subsequent divisions. Cells with a longer doubling time (green) also had long intervals between subsequent divisions. The reason for such an ‘inheritance’ is unknown.

### Asymmetric division under glucose starvation

**Fig. 3:**
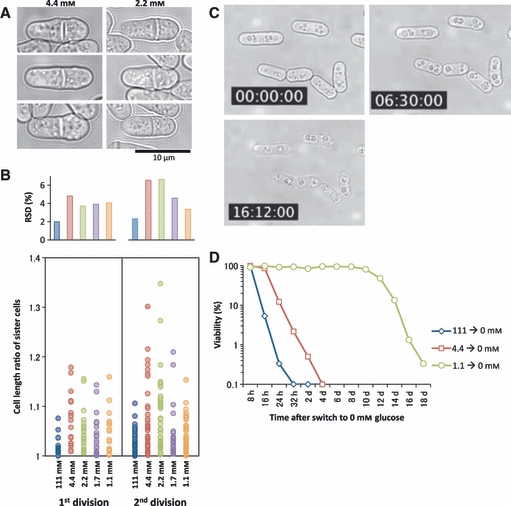
Asymmetric division and lifespan increase of cells in 0 mm glucose that had been treated previously by starvation (A) Representative micrographs of cells that display asymmetric septation in medium containing 4.4 or 2.2 mm glucose. (B) Cell length ratio of two daughter cells from one mother is shown in the first and second divisions for different glucose concentrations. In the top section, the relative standard deviation (RSD) of the ratios is plotted. (C) Images from movies of cells in the medium lacking glucose (fasting condition). The number indicates time (h:min:s). (D) Lifespan increase for the cells pretreated by glucose starvation. Viability (%) was measured for cells shifted from the culture containing excess glucose (111 mm) directly to fasting glucose (0 mm; blue line) and for cells previously treated by diet glucose (4.4 mm; red) or starvation glucose (1.1 mm; green) for 16 h.

The division pattern of *S. pombe* is symmetrical with regard to the site of septation and cytokinesis. The positions of septation were often not precisely at the equator in dividing cells in 4.4 and 2.2 mm glucose (Fig. 3A). The relative standard deviation of the cell length ratio of the daughter cells (unity indicates perfectly symmetrical division) was about 2% in 111 mm glucose, but increased to 3–6% at glucose concentrations below 4.4 mm (Fig. 3B). It should be noted that cell viability did not decrease, even in 1.1 mm glucose; thus, these asymmetric divisions apparently do not affect viability.

### Fasting causes the arrest of organelle movement and the loss of viability

When shifted to a 0 mm glucose medium (i.e. ‘fasting’), cell cycle progression was immediately blocked (Movie S1). A small increase (< 1%) in the cell number, however, was observed; a tiny fraction of cells with a septum appeared to commit cell separation even after the initiation of fasting. The movement of intracellular organelles was arrested around 1–2 h after the initiation of fasting. Significant changes were observed in the cytoplasmic features (e.g. large, apparently empty vacuoles) after 6 h (Fig. 3C). Cells displaying these striking changes were still viable, as their ability to form colonies on a replenished glucose-containing plate was nearly 100% after 8 h in 0 mm glucose. Cell viability after the abrupt shift to the 0 mm glucose liquid culture from the standard 111 mm glucose medium was found to be nearly completely lost, however, after 32 h (Fig. 3D; blue line).

### Previous starvation increases lifespan under fasting

The lifespan of cells under 0 mm glucose was prolonged if the cells were precultured under starvation conditions. When cells were precultured in 4.4 mm glucose (diet condition) for 16 h and then shifted to 0 mm glucose, viability improved slightly from 32 h to 2–4 days (Fig. 3D; red line). If precultured in 1.1 mm glucose (starvation condition) for 16 h and then changed to 0 mm glucose, the cell lifespan was dramatically prolonged (green line). Viability remained over 90% and 81% for 8 and 10 days, respectively, and then decreased to 1% at 16 days. Previous starvation treatment thus increased the lifespan by approximately 10 times under the fasting condition. These remarkable findings of a lifespan increase under fasting conditions by previous starvation were further investigated by metabolomic analysis (see below).

### Metabolic biomarkers revealed under different glucose concentrations

We evaluated the cellular metabolic changes that occurred on changes in the glucose concentration. Metabolic profiling was performed using LC/MS as described previously [23]. Methanol (50%)-extracted samples obtained from *S. pombe* wild-type cells grown in liquid culture containing different glucose concentrations for 6 h at 26 °C were analyzed. It should be noted that the glucose concentrations below are the initial values, and culture conditions cannot be considered to be completely steady as in the case of the perfusion system; the actual glucose concentrations decreased at the time (6 h) of metabolite extraction, but were very dependent on the cell concentrations. For the case of 4.4 mm glucose and the initial cell concentration of 2 × 10^6^ mL^−1^, a concentration of ∼3 mm glucose remained at the time (6 h) of metabolite extractions (Fig. 1A). The numerical results of three sample extractions in each condition are reported in Table S1. The results of independent metabolomic experiments were mostly reproducible.

#### ATP, ADP, AMP and adenosine

**Fig. 4:**
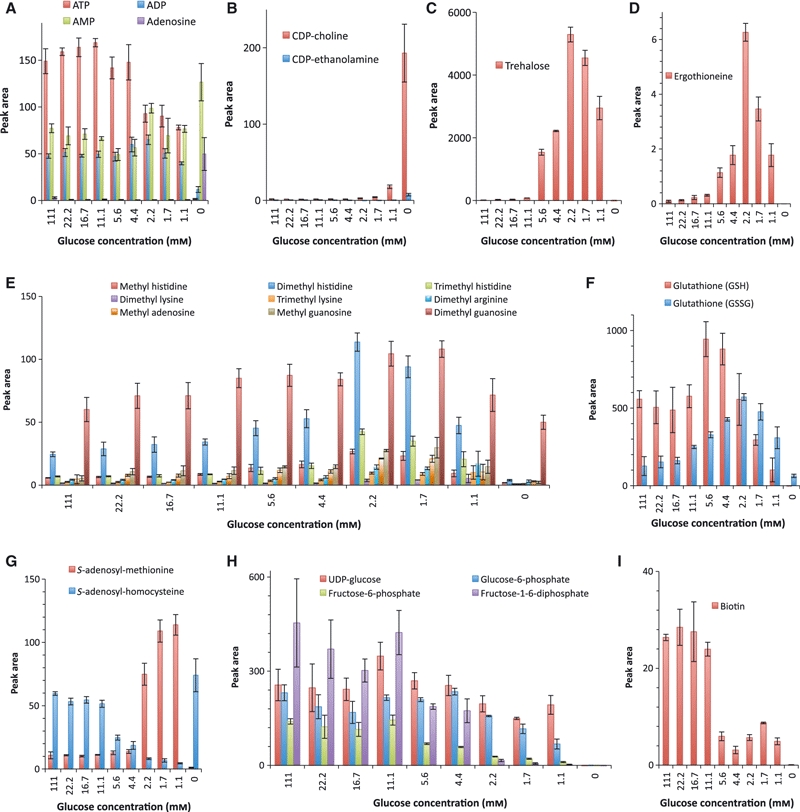
Peak areas of potential biomarker compounds in 10 different glucose concentrations determined by the LC/MS method. Cells were switched to media containing 10 different glucose concentrations and cultivated for 6 h. Note that the glucose concentrations were initial at the start of cultivation. Metabolite extracts were prepared three times and mean peak areas with standard deviations of the following metabolites are shown: (A) ATP, ADP, AMP and adenosine; (B) CDP-choline, CDP-ethanolamine; (C) trehalose; (D) ergothioneine; (E) methylated amino acids and nucleosides; (F) reduced (GSH) and oxidized (GSSG) glutathione; (G) *S*-adenosyl-methionine, *S*-adenosyl-homocysteine; (H) UDP-glucose, glucose-6-phosphate, fructose-6-phosphate, fructose-1,6-diphosphate; (I) biotin.

ATP levels were high in 111 to 1.1 mm glucose, 6 h after the glucose shift (Fig. 4A). Under 0 mm glucose, ATP levels decreased dramatically, whereas AMP and adenosine increased sharply. GTP, CTP, UTP and phosphoenolpyruvate behaved similarly to ATP (Table S1). The high-energy compounds were thus plentiful, even in 1.1 mm glucose, but decreased strongly in 0 mm glucose.

#### Compounds decreased or increased in the fasting condition

Certain biosynthetic precursor compounds, such as UDP-glucose, acetyl-CoA and phosphoglyceric acid, were virtually absent in 0 mm glucose, like ATP and other high-energy compounds, but plentiful at higher glucose concentrations. In contrast, the CDP-bound lipid components, CDP-choline and CDP-ethanolamine (precursors for phosphatidylcholine and phosphatidylethanolamine, respectively), increased sharply (Fig. 4B).

#### Increase in ergothioneine and trehalose in low glucose

Two metabolic compounds showed sharply increased levels in low-glucose (1.1–5.5 mm) cultures. The peak area of trehalose, a disaccharide (α,α-1,1-glucoside bond between two α-glucose units), increased strongly in 2.2 mm glucose (Fig. 4C). Another increased compound, ergothioneine, is a trimethylated thiol derivative of histidine (Fig. 4D). Trimethyl histidine, a precursor of ergothioneine, also increased sharply in 2.2 mm glucose (Fig. 4E). It was noted that a number of methylated amino acids and nucleosides were also increased in low glucose (Fig. 4E). Trehalose and ergothioneine were produced in cells under the 5.6 mm glucose condition, whereas only small amounts were produced in the two-fold higher (11.1 mm) glucose condition, indicating that 5.6 mm was the threshold glucose concentration for the production of trehalose and ergothioneine.

The potent antioxidant glutathione, a tripeptide of glutamate, cysteine and glycine, was abundant at all glucose concentrations, except for 0 mm glucose (Fig. 4F). Oxidized glutathione, however, did not increase. Cells under abrupt fasting therefore seemed susceptible to oxidative stress. We encountered some technical difficulties with reproducible measurements of glutathione levels, so that a number of measurements were performed for glutathione.

#### Glycolysis-related metabolites

Glycolysis pathway intermediates, such as phosphorylated glucose and fructose, were abundant at high glucose concentrations, but diminished in the starvation condition and were absent in the fasting condition (Fig. 4H). UDP-glucose (activated form of glucose), however, maintained a high level, even in 1.1 mm glucose, and only disappeared in the fasting condition.

Fructose-1,6-diphosphate, an intermediate in glycolysis prior to cleavage into triose, decreased strongly at glucose concentrations below 11.1 mm. The change seemed to be the reverse of that of trehalose.

#### *S*-Adenosyl methionine and methylation products

*S*-Adenosyl-methionine (SAM), a methyl donor compound, increased strongly (∼20-fold) at glucose concentrations below 2.2 mm, whereas *S*-adenosyl-homocysteine (SAH) levels decreased. In the fasting condition, the SAM level was minimal, whereas the SAH level increased sharply (Fig. 4G). SAH may be a marker compound that increases during fasting, whereas SAM may be a marker metabolite that increases during starvation. The methyl transfer reactions to proteins such as histones and tRNAs might be activated under glucose starvation, but not in fasting.

#### Biotin

The level of biotin, which was high in excess (111 mm) and standard (11 mm) glucose, diminished in the diet and starvation conditions of glucose, and decreased to zero in the fasting condition (Fig. 4I). The changes in biotin were unique, as no other metabolite showed a similar pattern of change according to glucose concentrations.

### Decay of energy metabolites and cell death under 0 mm glucose

Following the abrupt transfer from 111 to 0 mm glucose for 6 h at 26 °C, the levels of energy-related metabolic compounds all became negligible (see above). All metabolites implicated in glycolysis and antioxidative stress-protective compounds decreased drastically. It should be noted that cells in 0 mm glucose for 6 h were unhealthy but not dead, as they could fully recover to form colonies if glucose was replenished at this time (Fig. 3D).

**Fig. 5:**
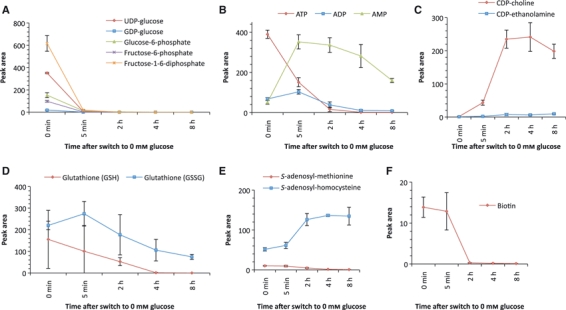
The time course change of the peak areas of key metabolites in cells switched to a fasting (0 mm) glucose condition from 111 mm glucose. *S. pombe* cells cultivated in mid-logarithmic phase (5 × 10^6^ cells·mL^−1^) in standard EMM2 medium containing 111 mm glucose were shifted to the fasting condition (0 mm glucose) and metabolites were extracted after 0 min (prior to shift), 5 min, 2 h, 4 h and 8 h. Three samples were prepared at each time point. Mean peak areas with standard deviations of the following metabolites are shown: (A) UDP-glucose, GDP-glucose, glucose-6-phosphate, fructose-6-phosphate, fructose-1,6-diphosphate; (B) ATP, ADP and AMP; (C) CDP-choline, CDP-ethanolamine; (D) reduced (GSH) and oxidized (GSSG) glutathione; (E) *S*-adenosyl-methionine, *S*-adenosyl-homocysteine; (F) biotin.

To determine how quickly the cells could respond to the fasting environment, the time course of changes of metabolites was analyzed (numerical data in Table S2). *S. pombe* cells first grown in 111 mm glucose were switched to 0 mm glucose, and metabolites were extracted at 0 and 5 min and 2, 4 and 8 h. Within 5 min, a large change occurred for many compounds. A very fast decay of UDP-glucose, phosphorylated glucose and fructose (Fig. 5A), phosphoenolpyruvate and acetyl-CoA (Table S2) was observed, indicating that the glycolysis pathway quickly consumed its remaining free intermediates. The loss of UDP-glucose within 5 min explains the lack of an increase in trehalose. ATP decreased by three-fold within 5 min (Fig. 5B), and AMP increased five- to six-fold. At 4 h, the level of ATP decreased to approximately 3%. CDP-ethanolamine and CDP-choline, markers for glucose fasting, began to increase at 5 min and increased steadily by 10- to 100-fold, respectively, at 8 h (Fig. 5C). Glutathione, SAM and biotin (Fig. 5D–F) levels decreased steadily to zero at around 8 h. Although the data for glutathione (GSH) were variable for unknown reasons, its mean peak area showed a clear decrease after 2 h. Taken together, the cell’s response to 0 mm glucose was very rapid, around 5 min, with regard to the shut-off of energy metabolism, but loss of viability occurred much later, after 8 h (Fig. 3D).

### Metabolic compound analysis during the lifespan increase after starvation

The increased lifespan after cells went through starvation was studied by analyzing the metabolites, and the results are shown in Fig. 6 and Table S3. Cells were cultured in 1.1 mm glucose medium for 7 days (1.1 mm was the initial concentration, and the exhaustion of glucose in the medium should occur within 1 day). Cells remained fully viable during the experiment (Fig. 6A). ATP levels decreased but, after 7 days, were still over 10 times higher than those of cells shifted immediately to 0 mm from 111 mm glucose (Fig. 6A). AMP levels were high after 2 and 7 days. The UTP, CTP and GTP levels after the starvation treatment were also much higher than those of cells transferred directly from 111 to 0 mm glucose (Table S3).

Anti-stress compounds, such as ergothioneine, its precursor trimethyl histidine and trehalose, were maintained at high levels in the starvation-mediated fasting condition (Fig. 6B, C). Levels of SAM and SAH were high after 7 days (Fig. 6D), suggesting the importance of this compound for longevity. The levels of CDP-choline (Fig. 6E) and ferrichrome (an iron-carrying compound; Fig. 6F) were high after 7 days.

### Oxidative stress and DNA damage signals in glucose-fasting and glucose-starved cells

**Fig. 7:**
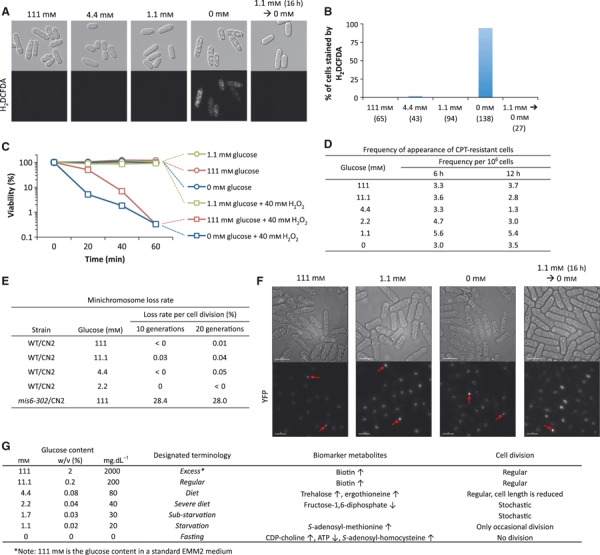
Oxidative stress and DNA damage observation. (A) Oxidative stress accumulation was observed using H_2_DCFDA dye. Cells were cultured for 6 h in 111, 4.4, 1.1 and 0 mm glucose, or precultivated in 1.1 mm glucose for 16 h prior to the switch to 0 mm glucose medium for 6 h (rightmost image). Micrographs show representative examples of cells. (B) Percentage of cells stained by H_2_DCFDA dye is plotted for each observed condition (see A). The number in parentheses indicates the amount of observed cells. (C) Cells were incubated in excess (111 mm), starvation (1.1 mm) and fasting (0 mm) glucose conditions for 6 h, followed by the addition of H_2_O_2_ to the final concentration of 40 mm. Viability (%) was measured in 20-min intervals. (D) The frequency of cells resistant to camptothecin (CPT) per 10^6^ cells was measured after 6 or 12 h of incubation in 111, 11.1, 4.4, 2.2, 1.1 and 0 mm glucose. (E) The rate of loss of minichromosome was measured using the CN2 strain [27] after incubation in 111, 11.1, 4.4 and 2.2 mm glucose. The *mis6-302* strain, which often loses a minichromosome [47], was used as the control. (F) Micrographs showing Rad22-YFP signals [45] after 8 h of cultivation in 111, 1.1 and 0 mm glucose. Red arrows indicate the observed Rad22 foci. The rightmost image shows cells precultivated in 1.1 mm glucose for 16 h prior to the switch to 0 mm glucose medium for 8 h. (G) Summary of the glucose concentrations described in this article. Concentrations are listed in three commonly used notations: millimolar (mm), percentage (w/v) or mg·dL^−1^. Biomarker metabolites increased (↑) or decreased (↓) in each condition are noted, as well as the corresponding cell division phenotypes.

Considering the rapid decrease in the antioxidants glutathione and ergothioneine in 0 mm glucose, we employed a fluorescent probe, 2′,7′-dichlorodihydrofluorescein diacetate (H_2_DCFDA), to check for the presence of oxidative stress. Only cells abruptly shifted from 111 to 0 mm glucose for 6 h showed strong fluorescent signals (Fig. 7A). We counted the percentage of cells stained by H_2_DCFDA in each condition (Fig. 7B). Although 94% of cells in 0 mm glucose were stained, almost no signals were observed in cells shifted from 111 mm to low glucose levels (1.1–4.4 mm) or in cells first treated with 1.1 mm glucose for 16 h and then transferred to fasting for 6 h. We interpret these results to indicate that the oxidative stress produced was not reduced appropriately in fasting cells as a result of the loss of antioxidant compounds. It should be noted that the cell viability was still nearly 100% at this time point (Fig. 3D).

The increase in ergothioneine in starvation conditions may indicate increased resistance to oxidative stress. We challenged the cells with 40 mm H_2_O_2_, a concentration previously reported to kill *S. pombe* cells within 1 h [24]. The results shown in Fig. 7C indicate that cells incubated in 0 mm glucose for 6 h (blue squares) were sensitive to H_2_O_2_ oxidative stress, whereas cells cultivated in 1.1 mm glucose (green squares) were much more resistant than cells in 111 mm glucose (red squares) or 0 mm fasting cells. Fasting cells abruptly shifted from 111 mm glucose were the most sensitive to 40 mm H_2_O_2_. These results are consistent with a very recent report showing that glucose starvation causes activation of the Sty1-dependent stress response pathway, which activates antioxidant production and increases resistance to stresses [25].

We further tested whether mutations, such as those resistant to camptothecin (CPT, an inhibitor of DNA topoisomerase) [26], arise frequently in cells under glucose starvation or fasting conditions. The number of CPT-resistant cells did not change significantly (Fig. 7D): 3.3–3.7 per 10^6^ cells in 111 mm glucose and 3.0–3.5 per 10^6^ cells in 0 mm glucose for 6 or 12 h. Similar results were obtained in cells in 1.1–4.4 mm glucose. We also tested the loss of minichromosomes using the CN2 strain [27], and found that cell division under 4.4 or 2.2 mm glucose did not affect the rate of chromosome loss compared with 111 mm glucose (Fig. 7E). Finally, we examined whether the yellow fluorescent protein (YFP) signals of the DNA strand break-sensitive protein Rad22 tagged with YFP formed frequent foci under starvation or fasting conditions. No dramatic increase in Rad22 foci was observed (Fig. 7F; cells displaying foci are indicated by red arrows).

## Discussion

*Schizosaccharomyces pombe* is widely distributed on Earth [28,29] and natural isolates were obtained from a variety of substrates, including millet beer (*pombe* means beer in Swahili). Its natural, ecological environment, however, is little known. Its actual living conditions in nature, particularly with regard to nutritional conditions, may be very diverse, from harsh conditions that would cause the arrest of cell division, to rich conditions that could support the cycles of rapid cell division. It is unknown what nutritional conditions are ‘optimum’ for this organism in nature. We were thus interested in how *S. pombe* responded to the culture conditions of limited glucose, and initiated the present study to analyze the division patterns under a series of glucose concentrations. For this analysis, we employed a microfluidic perfusion system, which ensured the constant supply of fresh medium in the microscope chamber.

An initial finding of the present study, that the doubling time of *S. pombe* was identical between 111 and 4.4 mm glucose, was surprising. Judging from the identical doubling time, these very different glucose concentrations seem to be optimal for *S. pombe* cell division. We therefore assumed that *S. pombe* may be used as a model of mammalian body cells that take up glucose from blood: 4.4 mm glucose is a pre-breakfast level in human blood. The reduction in cell size might at least partly explain the short doubling time in low glucose. The 33% reduction in cell length might reduce the duration of the cell division cycle. The size control for mitotic entry might thus be altered under glucose limitation. A protein kinase Ssp1, similar to calcium–calmodulin-dependent protein kinase kinase, may be implicated in this control, as *ssp1* mutant cells are elongated under limited glucose concentrations [30]. A systematic genetic screen was performed to isolate a number of mutants that were unable to support cell division below 4.4 mm glucose concentration, and more than 100 mutant genes were identified. These genes were mostly conserved in eukaryotes (S. Saitoh *et al.*, unpublished results).

The regularity of cell division timing seemed to be disrupted under glucose starvation, and the critical transition for division and quiescence occurred between the severe diet (2.2 mm) and substarvation (1.7 mm) conditions. Based on the cellular and metabolic phenotypes obtained, we named the different glucose concentrations excess (111 mm), regular (11.1 mm), diet (4.4 mm), severe diet (2.2 mm), substarvation (1.7 mm), starvation (1.1 mm) and fasting (0 mm), as summarized in Fig. 7G. These terminologies for *S. pombe* bear some resemblance to the terminology used for human blood sugar content, but we do not intend to imply that they have any direct relationship. The terminologies for *S. pombe* should be restricted to use only for the nutritional conditions of *S. pombe*. In humans, symptoms of hypoglycemia develop at 2.7 mm blood glucose, and seizures may occur as glucose falls to 1.7 mm. These concentrations correspond to the *S. pombe* terminologies of severe diet and substarvation, respectively. When blood glucose levels fall below 0.5 mm, human neurons are no longer functional, resulting in coma. In *S. pombe*, cell division is mostly arrested, but it does not lose viability in 1.1 mm glucose. It remains to be determined whether very rapid responses and changes in important metabolites in *S. pombe* after the change in glucose concentrations have any parallel to the events that occur in human body cells.

We were able to identify metabolic biomarkers for different glucose concentrations, such as biotin (excess), ergothioneine (diet), SAM (starvation) and CDP-choline (fasting). The identification of marker metabolites up- or down-regulated in a particular cellular condition may indicate which pathways play an important role in the maintenance of *S*. *pombe* cells in that glucose condition, and assist in the characterization of other conditions in future research by comparing their levels. High-energy phosphate compounds might represent metabolic markers for healthy viable cells, even under severe starvation. Their rapid decay was only observed in cells after an abrupt shift to glucose fasting. The phospholipid-related CDP compounds dramatically increased under the 0 mm glucose condition. The reason for the increase in these compounds remains to be clarified: one possible reason is a cellular attempt to create membranous structures under the fasting condition. Alternatively, the conversion from the precursors to phospholipids may be blocked in the fasting condition. In any case, CDP-choline and CDP-ethanolamine are not death marker compounds, as glucose-fasted cells at the time of their sharp increase were fully viable.

Trehalose protects proteins and membranes from inactivation or denaturation [31]. In *S. pombe*, trehalose is produced under various physiologic stresses [32]. Ergothioneine, an antioxidant, increased, possibly as a result of the activation of mitochondria that produce oxidative stress [33]. It increased in the 4.4 mm diet condition, when the doubling time was 3.5 h, similar to that in excess glucose, suggesting that diet cells might be under oxidative stress, although the rate of cell division was the most rapid. Ergothioneine in humans has been reported to act as an antioxidant compound with a role against inflammation, implicated in rheumatoid arthritis and Crohn’s disease [34]. Ergothioneine is present in human blood, and is considered to be important for the protection of mitochondrial activity [35]. The threshold glucose concentration (5.6 mm) for the production of trehalose and ergothioneine in *S. pombe* may coincide with the intensified generation of oxidative stress by mitochondria and other cells. Starvation might produce highly protective cells by increasing the levels of these compounds.

There is no obvious explanation why SAM increased under severe diet and starvation conditions. Methylation might play an important role in cells under low glucose. It is possible that the increase in methylation, such as for histones [36], might diminish gene transcription, which would cause slow division or quiescence. Further study is necessary. Fructose-1,6-phosphate decreased strongly under glucose starvation, possibly as a result of the increase in fructose-1,6-diphosphatase enzyme under glucose limitation [37]. Fructose-1,6-diphosphatase is a well-known glucose-repressing enzyme, and catalyzes fructose-1,6-diphosphate to fructose-6-phosphate. The level of fructose-6-phosphate was still high under the 4.4 and 2.2 mm glucose conditions. Fructose-1,6-diphosphate may be considered as a decreasing marker metabolite under diet and starvation conditions, which is consistent with the large increase in fructose-1,6-diphosphatase.

Biotin, a vitamin that represents the activity of sugar, fatty acid and amino acid metabolism, was a unique marker metabolite for growth (cell size) and division. It was found to be abundant in maximally growing *S. pombe* cells under excess glucose content, diminished in short-sized cells under diet and starvation, and was virtually absent in the growth-arrested fasting condition. Biotin is a vitamin bound to various carboxylases, including key enzymes such as pyruvate carboxylase and acetyl-CoA carboxylase, which control sugar and fatty acid metabolism [38]. The level of biotin might correlate in a certain way with the cellular ability to utilize sugar and fatty acid.

Previously, we have reported two genes that are required to properly utilize the diet glucose level (4.4 mm), but are not needed under excess glucose conditions. They encode Ssp1 kinase (homolog of calcium–calmodulin-dependent protein kinase kinase) and Sds23 (an inhibitor of type 2A-related protein phosphatases including Ppe1) [30]. Ssp1 and Sds23 are functionally closely related as the phenotype of *ssp1* kinase mutants was suppressed by the increased gene dosage of Sds23, and vice versa. Glucose consumption was decreased significantly in these mutants, suggesting that the import or actual consumption of glucose might be impaired. Ssp1 kinase is involved in the G2/M transition [39], so that glucose metabolism may be related to cell cycle regulation. In humans, calcium–calmodulin-dependent protein kinase kinase has a role in utilizing glucose through interaction with AMP-dependent protein kinase [40], which is implicated in diabetes [41]. In *S. pombe*, Ssp2 is the AMP-dependent protein kinase, and Ssp1 and Ssp2, whose mutants show similar phenotypes, interact closely [30,39].

*Schizosaccharomyces pombe* responds very differently to nitrogen and glucose starvation [19]. In the absence of a nitrogen source, cells undergo meiosis if a sexual partner cell is nearby. If not, cells become completely arrested after two rounds of cell division without cell growth, and the resulting small, round cells remain viable for months, as they possess the ability to reuse intracellular nitrogen [42]. The nitrogen source-starved quiescent G0 cells contain prereplicative 1C DNA, are efficient in DNA damage repair and active in various metabolic cellular pathways [43]. On replenishment of the nitrogen source, cells undergo the first mitosis after cell growth and DNA replication. In glucose fasting, however, *S. pombe* immediately stops cell division and loses viability within 32 h. These glucose-fasting arrested cells are rod-shaped and contain postreplicative 2C chromosomal DNA (*S. pombe* growing cells are mostly in the G2 phase). They are not a typical form of quiescence as they are devoid of ATP and lose their viability fairly quickly. However, cells subjected to glucose fasting after starvation pretreatment contain a high level of ATP and show more than a 10-fold increase in the chronological lifespan. Hence, these glucose-fasting quiescent cells after starvation pretreatment are worth investigating in detail. In these long-lived quiescent cells, high-energy compounds and anti-stress compounds are abundant. Starvation pretreatment might produce highly protective cells by increasing the levels of stress-responsive compounds, together with a large increase in protective compounds, such as trehalose. The high levels of nucleotide triphosphates and other high-energy compounds may not be sufficient to explain why cells pretreated with 1.1 mm glucose were able to live so long. Additional properties, such as stress resistance and stress protection, might be equally or more important for their longevity. It should be noted that our results also indicate that the loss of viability as a result of DNA damage was negligible under fasting or starvation conditions.

Variations in the mode of cell division became apparent under diet glucose conditions. Division timing and symmetry seemed to become less uniform under limited glucose, suggesting that unknown factors related to nutrition affect the uniformity of division. The irregularities of division may be understood if we assume that nutrition is insufficient or in short supply. We asked the question of what was actually inherited from mother to daughter to keep the generation time the same under low glucose. We speculate that the ‘inheritance’ of nongenetic materials or epigenetic properties from the mother cells influences the doubling time of daughters for a few generations. The stochastic switching between proliferation and quiescence might be a result of variations in the abundance of organelles or the degree of cellular aging, or epigenetic differences caused by various metabolic and cellular structural differences. Glucose starvation might enhance such differences. In other words, the evolution of *S. pombe* may occur more rapidly under diet to starvation conditions, as cellular variations were more prominent than in glucose-rich medium.

## Materials and methods

### Strains and growth conditions

The wild-type heterothallic haploid 972 h^−^*S. pombe* strain [44] was used for the metabolomic and viability experiments. Cells were cultivated in minimal synthetic medium EMM2 [5,7] with modified glucose content. Limited glucose media were prepared by mixing regular EMM2 (2% glucose) medium with EMM2-G (0% glucose) in the appropriate ratio. The cultivation temperature was 26 °C unless otherwise stated. A haploid h^+^*rad22-YFP:KanR* strain, obtained by back-crossing the EN3222 strain [45] with a wild-type 975 h^+^ strain, was used to observe the Rad22 foci. The CN2 strain carrying an artificial linear minichromosome, Ch10 (*sup3-5*), in the *ade6-704* background [27] was used for the minichromosome loss assay, and *mis6-302* [46,47] strain was used as the control.

### Culture glucose measurement

Cells were cultivated in 111 mm glucose medium to mid-logarithmic phase (5 × 10^6^ cells·mL^−1^), and then placed in 4.4 mm glucose or 111 mm glucose (control) medium at a concentration of 2 × 10^6^ cells·mL^−1^ after washing. Samples of 500 μL were taken at each time point to measure the concentrations of cells and glucose. The glucose concentration was determined using the Glucose (HK) assay kit (Sigma-Aldrich, St. Louis, MO, USA), following the manufacturer’s instructions.

### Microscopy and movies

Cells were cultivated in 111 mm glucose medium to logarithmic phase (2 × 10^6^ cells·mL^−1^), and then fixed in a microscopic specimen chamber that was continuously supplied with culture medium (Onix™ Microfluidic Perfusion System) at a flow rate of 3 μL·h^−1^. The temperature in the room was set to 26 °C. Following the medium change to 111 (control), 11.1, 4.4, 2.2, 1.7, 1.1 or 0 mm glucose medium, photographs were taken every 3 min using a DeltaVision microscope system (Applied Precision), and movies were created from the photographs. The cell length was measured using Adobe Photoshop from the micrographs. Microscopic observation was repeated three times with similar results.

### Viability measurement

Cell viability was measured by plating 300 cells on a YPD (1% yeast extract, 2% polypeptone and 2% glucose) agar plate, incubating the plate at 26 °C for several days, and counting the number of colonies formed. Viability was calculated as the percentage of the number of formed colonies against 300.

### Sample preparation for metabolomic analysis

Samples were prepared as described previously [23]. Cells from cultures (40 mL per sample, 5 × 10^6^ cells·mL^−1^) were collected by vacuum filtration and immediately quenched in –40 °C methanol. Cells were harvested by centrifugation and constant amounts of internal standards (10 nmol of HEPES and PIPES) were added to each sample. Cells were disrupted using a Multi-Beads Shocker (Yasui Kikai, Osaka, Japan) in 500 μL of 50% methanol. Proteins were removed by filtering on an Amicon Ultra 10-kDa cut-off filter (Millipore, Billerica, MA, USA) and samples were concentrated by vacuum evaporation. Finally, each sample was resuspended in 20 μL of 50% acetonitrile and 1 μL was used for autosampler injection.

### LC-MS analysis

LC-MS data were obtained using a Paradigm MS4 HPLC system (Michrom Bioresources, Auburn, CA, USA) coupled to an LTQ Orbitrap mass spectrometer (Thermo Fisher Scientific, Waltham, MA, USA). LC separation was performed on a ZIC-pHILIC column (Merck SeQuant, Umeå, Sweden; 150 mm × 2.1 mm, 5 μm particle size). Acetonitrile (A) and 10 mm ammonium carbonate buffer, pH 9.3 (B) were used as the mobile phase, with gradient elution from 80% A to 20% A in 30 min, at a flow rate of 100 μL·min^−1^. The peak areas of the metabolites of interest were measured using MZmine 2 software [48] and normalized by the peak areas of the spiked internal standards. Detailed data analysis procedures and parameters have been described previously [23].

### Oxidative stress staining

The procedure described previously [49] was followed. Cells were incubated with H_2_DCFDA dye (Invitrogen, Carlsbad, CA, USA; final concentration 10 μg·mL^−1^) for 80 min in the absence of light, and then washed twice with 50 mm sodium citrate (pH 7.0) and kept on ice until observation. Images were obtained using an AxioPlan 2 (Carl Zeiss AG, Oberkochen, Germany) microscope.

### H_2_O_2_ resistance assay

A 4 m stock solution of H_2_O_2_ was prepared in H_2_O. Cells were cultivated in excess (111 mm), starvation (1.1 mm) and fasting (0 mm) glucose conditions for 6 h (3 × 10^6^ cells·mL^−1^), followed by the addition of H_2_O_2_ to a final concentration of 40 mm, as described previously [24]. Viability in the presence and absence of H_2_O_2_ was measured for 1 h in 20-min intervals (see Viability measurement section).

### CPT resistance assay

Cells were incubated for 6–12 h in low-glucose medium at 26 °C, and then incubated for 18 h in 111 mm glucose medium at 26 °C. After the cell number had increased over 10-fold, cells were plated on YPD plates with or without 25 μm CPT. After incubation at 36 °C for several days, the number of colonies was counted.

### Minichromosome loss assay

CN2 cells [27] were cultured in EMM2 medium supplemented with leucine, and then diluted in low-glucose medium supplemented with leucine and adenine. Following growth for 10 or 20 generation times at 26 °C, cells were plated on YPD plates and incubated at 26 °C. Total colonies and red colonies were counted, and the percentage mitotic loss rates were calculated as described previously [50].

### Rad22 foci observation

*Rad22-YFP* strain was used to observe Rad22 foci as described previously [45]. Cells were fixed by methanol (−80 °C), washed three times in NaCl/P_i_ buffer, and kept on ice prior to observation. Images were obtained using a DeltaVision microscope (Applied Precision). Ten *z*-axis sections at 0.3-μm intervals were scanned and projected on a two-dimensional plot.

## Abbreviations

CPT: camptothecin
HEPES: 4-(2-hydroxyethyl)-1-piperazineethanesulfonic acid
H_2_DCFDA: 2′,7′-dichlorodihydrofluorescein diacetate
PIPES: piperazine-N,N′-bis(2-ethanesulfonic acid)
SAH: *S*-adenosyl-homocysteine
SAM: *S*-adenosyl-methionine
YFP: yellow fluorescent protein

## Supporting information

The following supplementary material is available:

**Fig. S1.** Cell behavior of *S. pombe* under limited glucose concentrations at 30 °C.

**Table S1.** Metabolic compounds from cell extracts obtained from *S. pombe* cells cultured in synthetic medium EMM2 containing 111, 22.2, 16.7, 11.1, 5.6, 4.4, 2.2, 1.7, 1.1 or 0 mm glucose for 6 h at 26 °C.

**Table S2.** Time course change in peak areas of metabolites under glucose fasting.

**Table S3.** Time course change in peak areas of metabolites under glucose starvation.

**Movie S1.** Cells after shifting to the ‘fasting’ condition (0 mm glucose).

**Movie S2.** Cells after shifting to the ‘starvation’ condition (1.1 mm glucose).

**Movie S3.** Cells after shifting to the ‘substarvation’ condition (1.7 mm glucose).

**Movie S4.** Cells after shifting to the ‘severe diet’ condition (2.2 mm glucose).

**Movie S5.** Cells after shifting to the ‘diet’ condition (4.4 mm glucose).

**Movie S6.** Cells after shifting to the ‘regular’ glucose condition (11.1 mm glucose).

**Movie S7.** Cells grown in culture containing ‘excess’ (111 mm) glucose.

This supplementary material can be found in the online version of this article.

Please note: As a service to our authors and readers, this journal provides supporting information supplied by the authors. Such materials are peer-reviewed and may be re-organized for online delivery, but are not copy-edited or typeset. Technical support issues arising from supporting information (other than missing files) should be addressed to the authors.

## References

[1] Saltiel AR, Kahn CR (2001). Insulin signalling and the regulation of glucose and lipid metabolism. Nature.

[2] Le T, Bhushan V, Vasan N (2010). First Aid for the USMLE Step 1.

[3] Nathan DM, Davidson MB, DeFronzo RA, Heine RJ, Henry RR, Pratley R, Zinman B (2007). Impaired fasting glucose and impaired glucose tolerance – implications for care. Diabetes Care.

[4] Leupold U (1970). Genetical methods for *Schizosaccharomyces pombe*. Methods Cell Physiol.

[5] Mitchison J (1970). Physiological and cytological methods for *Schizosaccharomyces pombe*. Methods Cell Physiol.

[6] Egel R (2004). The Molecular Biology of Schizosaccharomyces pombe: Genetics, Genomics and Beyond.

[7] Nurse P (1975). Genetic control of cell size at cell division in yeast. Nature.

[8] Yamamoto M (1996). Regulation of meiosis in fission yeast. Cell Struct Funct.

[9] Hagan I (1998). The fission yeast microtubule cytoskeleton. J Cell Sci.

[10] Yanagida M (2005). Basic mechanism of eukaryotic chromosome segregation. Philos Trans R Soc B Biol Sci.

[11] Rowley R, Hudson J, Young PG (1992). The wee1 protein kinase is required for radiation-induced mitotic delay. Nature.

[12] Kelly TJ, Martin GS, Forsburg SL, Stephen RJ, Russo A, Nurse P (1993). The fission yeast cdc18+ gene product couples S phase to START and mitosis. Cell.

[13] Bahler J (2005). A transcriptional pathway for cell separation in fission yeast. Cell Cycle.

[14] Grewal SI (2000). Transcriptional silencing in fission yeast. J Cell Physiol.

[15] Schafer B (2003). Genetic conservation versus variability in mitochondria: the architecture of the mitochondrial genome in the petite-negative yeast *Schizosaccharomyces pombe*. Curr Genet.

[16] Takegawa K, Iwaki T, Fujita Y, Morita T, Hosomi A, Tanaka N (2003). Vesicle-mediated protein transport pathways to the vacuole in *Schizosaccharomyces pombe*. Cell Struct Funct.

[17] Jourdain I, Sontam D, Johnson C, Dillies C, Hyams JS (2008). Dynamin-dependent biogenesis, cell cycle regulation and mitochondrial association of peroxisomes in fission yeast. Traffic.

[18] Roux AE, Chartrand P, Ferbeyre G, Rokeach LA (2010). Fission yeast and other yeasts as emergent models to unravel cellular aging in eukaryotes. J Gerontol A Biol Sci Med Sci.

[19] Yanagida M (2009). Cellular quiescence: are controlling genes conserved?. Trends Cell Biol.

[20] Roux AE, Leroux A, Alaamery MA, Hoffman CS, Chartrand P, Ferbeyre G, Rokeach LA (2009). Pro-aging effects of glucose signaling through a G protein-coupled glucose receptor in fission yeast. PLoS Genet.

[21] Madigan M, Martinko J, Dunlap P, Clark D (2008). Brock Biology of Microorganisms.

[22] Dulbecco R, Freeman G (1959). Plaque production by the polyoma virus. Virology.

[23] Pluskal T, Nakamura T, Villar-Briones A, Yanagida M (2010). Metabolic profiling of the fission yeast *S. pombe*: quantification of compounds under different temperatures and genetic perturbation. Mol Biosyst.

[24] Lee J, Dawes IW, Roe JH (1995). Adaptive response of *Schizosaccharomyces pombe* to hydrogen peroxide and menadione. Microbiology.

[25] Zuin A, Carmona M, Morales-Ivorra I, Gabrielli N, Vivancos AP, Ayte J, Hidalgo E (2010). Lifespan extension by calorie restriction relies on the Sty1 MAP kinase stress pathway. EMBO J.

[26] Eng WK, Faucette L, Johnson RK, Sternglanz R (1988). Evidence that DNA topoisomerase I is necessary for the cytotoxic effects of camptothecin. Mol Pharmacol.

[27] Niwa O, Matsumoto T, Chikashige Y, Yanagida M (1989). Characterization of *Schizosaccharomyces pombe* minichromosome deletion derivatives and a functional allocation of their centromere. EMBO J.

[28] Schlake T, Gutz H (1993). Mating configurations in *Schizosaccharomyces pombe* strains of different geographical origins. Curr Genet.

[29] Patch AM, Aves SJ (2007). Fingerprinting fission yeast: polymorphic markers for molecular genetic analysis of *Schizosaccharomyces pombe* strains. Microbiology.

[30] Hanyu Y, Imai KK, Kawasaki Y, Nakamura T, Nakaseko Y, Nagao K, Kokubu A, Ebe M, Fujisawa A, Hayashi T (2009). *Schizosaccharomyces pombe* cell division cycle under limited glucose requires Ssp1 kinase, the putative CaMKK, and Sds23, a PP2A-related phosphatase inhibitor. Genes Cells.

[31] Elbein AD, Pan YT, Pastuszak I, Carroll D (2003). New insights on trehalose: a multifunctional molecule. Glycobiology.

[32] Cansado J, Vicente-Soler J, Soto T, Fernandez J, Gacto M (1998). Trehalose-6P synthase is essential for trehalase activation triggered by glucose, nitrogen source or heat shock, but not by osmostress, in *Schizosaccharomyces pombe*. Biochim Biophys Acta.

[33] Wallace DC (1999). Mitochondrial diseases in man and mouse. Science.

[34] Grundemann D, Harlfinger S, Golz S, Geerts A, Lazar A, Berkels R, Jung N, Rubbert A, Schomig E (2005). Discovery of the ergothioneine transporter. Proc Natl Acad Sci USA.

[35] Paul BD, Snyder SH (2009). The unusual amino acid L-ergothioneine is a physiologic cytoprotectant. Cell Death Differ.

[36] Grewal SI, Rice JC (2004). Regulation of heterochromatin by histone methylation and small RNAs. Curr Opin Cell Biol.

[37] Vassarotti A, Friesen JD (1985). Isolation of the fructose-1,6-bisphosphatase gene of the yeast *Schizosaccharomyces pombe.* Evidence for transcriptional regulation. J Biol Chem.

[38] Pacheco-Alvarez D, Solorzano-Vargas RS, Del Rio AL (2002). Biotin in metabolism and its relationship to human disease. Arch Med Res.

[39] Matsusaka T, Hirata D, Yanagida M, Toda T (1995). A novel protein kinase gene ssp1+ is required for alteration of growth polarity and actin localization in fission yeast. EMBO J.

[40] Witczak CA, Sharoff CG, Goodyear LJ (2008). AMP-activated protein kinase in skeletal muscle: from structure and localization to its role as a master regulator of cellular metabolism. Cell Mol Life Sci.

[41] Zhang BB, Zhou G, Li C (2009). AMPK: an emerging drug target for diabetes and the metabolic syndrome. Cell Metab.

[42] Sajiki K, Hatanaka M, Nakamura T, Takeda K, Shimanuki M, Yoshida T, Hanyu Y, Hayashi T, Nakaseko Y, Yanagida M (2009). Genetic control of cellular quiescence in *S. pombe*. J Cell Sci.

[43] Mochida S, Yanagida M (2006). Distinct modes of DNA damage response in *S. pombe* G0 and vegetative cells. Genes Cells.

[44] Gutz H, Heslot H, Leupold U, Loprieno N (1974). Schizosaccharomyces pombe. Handb Genet.

[45] Noguchi E, Noguchi C, McDonald WH, Yates JR, Russell P (2004). Swi1 and Swi3 are components of a replication fork protection complex in fission yeast. Mol Cell Biol.

[46] Takahashi K, Yamada H, Yanagida M (1994). Fission yeast minichromosome loss mutants mis cause lethal aneuploidy and replication abnormality. Mol Biol Cell.

[47] Saitoh S, Takahashi K, Yanagida M (1997). Mis6, a fission yeast inner centromere protein, acts during G1/S and forms specialized chromatin required for equal segregation. Cell.

[48] Pluskal T, Castillo S, Villar-Briones A, Oresic M (2010). MZmine 2: modular framework for processing, visualizing, and analyzing mass spectrometry-based molecular profile data. BMC Bioinformatics.

[49] Marchetti MA, Weinberger M, Murakami Y, Burhans WC, Huberman JA (2006). Production of reactive oxygen species in response to replication stress and inappropriate mitosis in fission yeast. J Cell Sci.

[50] Murray AW, Szostak JW (1983). Pedigree analysis of plasmid segregation in yeast. Cell.

