# Supplementary material for: Specific biomarkers for stochastic division patterns and starvation-induced quiescence under limited glucose levels in fission yeast
Source: FEBS J. 2011 Apr;278(8):1299–315. doi: 10.1111/j.1742-4658.2011.08050.x (PMC3123465; doi:10.1111/j.1742-4658.2011.08050.x)
Supplement: Supplementary file 1 [file febs0278-1299-SD1.pdf]

the FEBS Journal

www.febsjournal.org

Specific biomarkers for stochastic division patterns and starvation-induced quiescence under limited glucose levels in fission yeast

Tomáš Pluskal, Takeshi Hayashi, Shigeaki Saitoh, Asuka Fujisawa and Mitsuhiro Yanagida

DOI: 10.1111/j.1742-4658.2010.08050

30°C

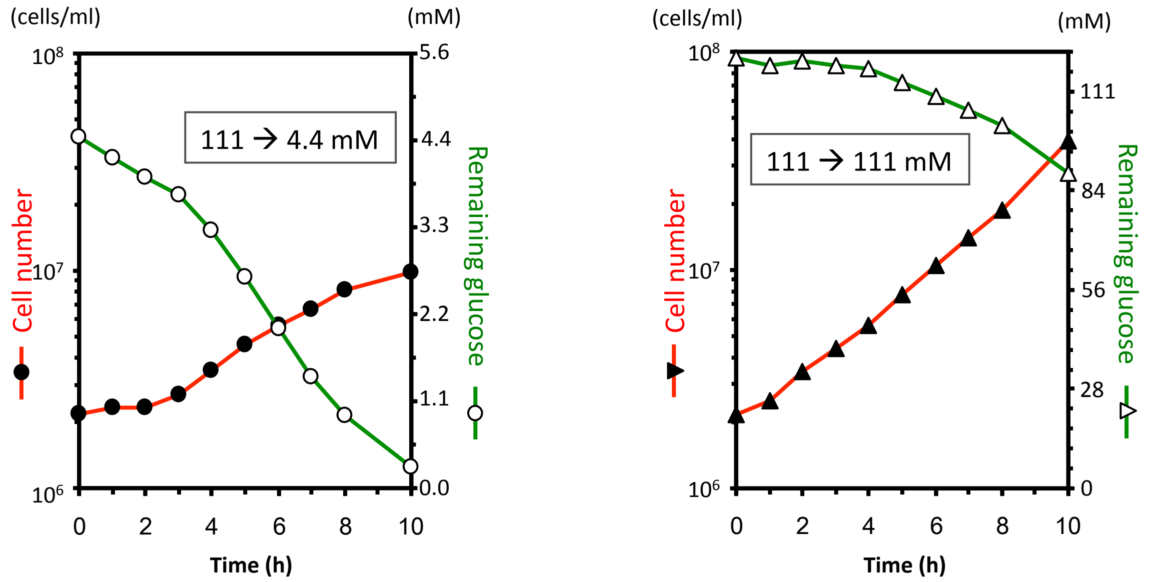

Figure S1. Cell behavior of *S. pombe* under limited glucose concentrations at 30°C
Cells cultured in standard medium containing 111 mM glucose were shifted to medium containing 4.4 mM glucose (left panel) or to control culture containing the same 111 mM glucose (right panel). The cell number increased and the glucose level remaining in the liquid culture were measured at 30°C for 10 h.

Ionization mode	m/z	Retention time (min)	Compound	Normalized peak area after 6 h cultivation in different glucose concentrations																													
				111 mM #1	111 mM #2	111 mM #3	22.2 mM #1	22.2 mM #2	22.2 mM #3	16.7 mM #1	16.7 mM #2	16.7 mM #3	11.1 mM #1	11.1 mM #2	11.1 mM #3	5.6 mM #1	5.6 mM #2	5.6 mM #3	4.4 mM #1	4.4 mM #2	4.4 mM #3	2.2 mM #1	2.2 mM #2	2.2 mM #3	1.7 mM #1	1.7 mM #2	1.7 mM #3	1.1 mM #1	1.1 mM #2	1.1 mM #3	0 mM #1	0 mM #2	0 mM #3
Positive	810.131	10.5	Acetyl-CoA	0.63	0.77	1.25	0.28	0.53	0.35	0.28	0.17	0.82	0.24	0.27	0.58	0.21	0.14	0.18	0.10	0.04	0.05	0.00	0.00	0.00	0.00	0.00	0.00	0.02	0.04	0.02	0.01	0.03	0.02
Positive	268.103	6.6	Adenosine	2.79	2.07	3.91	0.96	1.09	1.02	0.96	1.06	0.90	0.68	0.79	1.56	1.02	0.72	0.67	0.68	0.57	0.53	0.85	0.81	0.82	0.76	0.52	0.87	0.99	0.43	0.30	36.26	69.55	43.16
Positive	428.035	12.8	ADP	50.28	46.17	46.17	48.12	56.11	49.58	47.82	49.33	46.39	45.46	51.43	51.25	46.18	42.58	51.90	50.86	63.97	64.84	60.30	64.05	70.40	45.96	55.88	49.12	37.95	40.44	40.74	12.67	14.15	8.10
Positive	348.069	11.4	AMP	81.00	78.83	72.03	79.13	60.16	68.12	68.63	77.53	66.98	65.16	65.05	68.51	47.70	43.55	56.09	47.40	65.24	56.79	99.19	103.58	93.36	81.75	48.08	78.32	77.97	72.35	79.38	131.04	143.94	104.81
Positive	508.001	13.9	ATP	164.37	143.04	139.72	163.02	159.56	155.04	152.51	168.44	170.79	170.84	172.02	163.79	139.89	131.34	154.37	127.97	165.65	150.05	103.21	89.61	85.78	77.56	92.98	100.28	75.85	80.91	77.57	2.16	1.24	1.36
Positive	245.095	6.2	Biotin	25.68	26.49	26.96	32.08	28.73	24.63	25.25	34.54	22.88	24.66	22.31	24.90	6.21	4.95	6.71	3.48	3.65	2.24	6.31	5.70	5.08	8.70	8.90	8.56	5.72	4.44	4.58	0.06	0.09	0.06
Positive	489.113	13.0	CDP-choline	1.97	1.87	1.56	1.58	1.58	1.57	1.57	1.57	1.31	1.68	1.75	1.40	1.50	1.61	2.00	1.35	1.75	1.78	2.64	2.47	3.29	4.90	4.08	3.63	15.21	19.46	18.88	227.40	199.43	152.29
Positive	447.066	13.8	CDP-ethanolamine	0.45	0.37	0.34	0.56	0.52	0.54	0.56	0.64	0.48	0.66	0.74	0.74	0.39	0.42	0.48	0.34	0.35	0.36	0.11	0.11	0.14	0.22	0.24	0.24	0.44	0.53	0.43	7.83	8.90	5.54
Negative	481.978	15.6	CTP	28.18	21.99	14.99	21.41	28.51	14.71	19.36	25.72	18.74	36.02	30.58	35.45	46.08	39.92	45.65	53.96	50.63	58.74	42.66	56.47	47.44	56.17	45.67	50.27	44.00	59.53	28.40	0.00	0.00	0.00
Positive	203.150	20.7	Dimethyl arginine	4.72	3.81	4.47	4.55	4.43	3.77	4.01	4.45	4.03	4.81	4.47	4.82	5.48	4.68	5.96	5.43	7.48	6.74	12.78	14.26	16.16	14.39	12.29	13.61	10.54	28.46	12.03	3.07	4.19	2.34
Positive	312.129	10.0	Dimethyl guanosine	71.24	54.54	54.69	68.64	62.46	81.99	72.14	81.06	60.28	78.06	92.93	84.12	83.60	80.99	97.30	86.18	87.86	78.21	113.27	93.88	106.24	114.72	101.65	108.13	84.97	58.89	70.94	52.57	53.90	43.55
Positive	184.107	9.7	Dimethyl histidine	22.61	24.70	26.26	34.80	27.18	24.47	29.88	39.24	27.74	34.55	31.77	36.63	44.12	40.07	51.74	49.46	61.01	47.93	121.47	107.08	112.62	99.63	84.08	98.45	52.32	40.05	49.99	3.44	4.56	3.98
Positive	175.144	19.7	Dimethyl lysine	1.64	1.50	1.70	1.52	1.59	1.25	1.43	1.37	1.32	1.34	1.26	1.55	1.53	1.24	1.60	1.39	1.81	1.48	3.11	3.30	4.95	4.21	4.09	4.08	3.35	9.18	3.41	0.83	1.10	0.72
Positive	230.095	12.2	Ergothioneine	0.08	0.14	0.06	0.15	0.10	0.14	0.18	0.31	0.18	0.35	0.29	0.30	0.96	1.15	1.31	1.97	1.98	1.38	6.00	6.63	6.16	3.34	3.10	3.95	2.13	1.32	1.88	0.00	0.00	0.00
Positive	741.235	4.9	Ferrichrome	31.02	27.73	24.86	38.90	50.97	31.04	43.20	52.77	58.23	59.43	50.08	56.84	89.57	60.81	60.85	76.53	93.35	97.91	119.88	132.58	85.30	111.74	136.05	119.63	99.96	97.76	94.14	1.35	1.67	0.84
Negative	338.989	15.6	Fructose-1-6-diphosphate	603.31	432.79	324.51	373.77	461.47	275.68	313.09	332.27	261.32	380.01	384.80	504.45	183.12	181.34	197.28	168.10	139.11	214.26	12.06	19.55	14.44	8.53	3.96	5.08	2.41	3.91	1.40	0.01	0.00	0.00
Negative	259.022	13.4	Fructose-6-phosphate	149.16	138.17	132.39	123.10	159.65	86.08	104.34	139.91	99.13	145.25	127.77	159.56	66.37	68.74	71.52	58.84	60.11	56.54	28.96	26.75	27.80	22.88	19.36	21.01	8.59	12.21	10.50	0.10	0.03	0.07
Negative	604.072	15.4	GDP-glucose	18.25	14.82	10.93	18.29	23.27	12.97	16.14	21.06	16.32	30.42	26.23	29.44	31.73	30.00	33.25	34.56	33.26	38.75	18.48	25.44	20.30	23.22	18.13	19.85	28.57	35.66	18.94	0.02	0.00	0.02
Negative	259.022	14.2	Glucose-6-phosphate	260.52	214.70	218.57	196.02	219.58	144.90	155.23	208.47	142.15	216.28	206.52	223.89	205.11	206.30	216.22	244.94	223.64	235.96	157.68	159.02	155.09	132.92	103.67	112.22	68.05	83.49	51.87	0.25	0.17	0.33
Negative	306.076	11.9	Glutathione (GSH)	550.02	507.42	615.50	523.73	599.90	391.18	622.17	508.32	332.17	548.67	520.73	659.95	816.60	1029.22	987.08	875.91	780.65	983.93	564.28	718.26	385.11	257.16	324.10	303.38	190.02	49.85	64.64	0.06	0.21	0.05
Negative	611.147	15.0	Glutathione (GSSG)	189.87	120.02	68.90	141.19	194.82	122.17	141.48	183.23	159.68	257.20	237.84	253.78	315.47	318.07	349.49	439.38	416.35	426.83	547.26	576.98	590.07	537.06	446.65	444.14	296.20	384.11	245.20	64.50	51.12	73.23
Positive	258.109	12.3	Glycerophosphocholine	1344.24	1325.97	1367.27	1205.97	1335.77	1213.60	1245.24	1208.01	1278.69	1217.93	1121.13	1202.30	1051.60	946.08	1114.32	702.79	1306.06	1171.72	677.22	647.90	552.73	629.08	654.09	717.39	896.47	668.62	802.32	907.23	1036.07	740.73
Negative	214.049	13.1	Glycerophosphoethanolamine	13.56	16.68	22.71	8.96	21.81	5.47	9.90	8.93	12.12	12.79	9.55	20.81	14.08	7.86	11.74	6.05	11.91	12.13	5.04	4.80	4.21	6.38	5.09	5.81	6.84	10.53	6.31	17.52	23.77	23.90
Negative	333.059	13.5	Glycerophosphoinositol	14.05	11.13	12.60	9.49	13.08	6.46	8.72	9.91	7.77	11.37	9.31	12.71	6.54	7.41	6.33	4.23	5.98	6.20	2.64	2.87	2.45	3.93	3.78	3.32	1.80	2.69	1.15	14.56	12.96	15.80
Negative	521.985	16.4	GTP	124.61	78.81	49.57	80.15	93.73	57.58	79.61	91.88	62.94	131.67	102.69	139.99	134.19	118.72	139.56	161.17	151.62	182.32	100.69	162.23	120.67	132.63	98.32	114.72	103.76	139.84	70.47	0.25	0.14	0.44
Negative	267.073	8.4	Inosine	33.90	37.00	38.46	33.70	32.25	33.50	36.85	39.40	31.03	30.43	31.19	32.65	7.73	6.55	8.33	4.64	4.60	3.59	25.99	18.84	23.01	12.92	10.96	10.07	77.29	94.33	71.29	5.37	17.92	10.98
Positive	282.119	12.5	Methyl adenosine	8.21	1.20	4.84	8.50	8.00	6.92	6.31	8.56	8.07	9.38	6.38	5.67	10.12	13.68	11.70	9.48	12.14	11.11	21.54	20.76	21.15	19.07	19.70	24.13	17.05	4.73	9.48	3.54	3.01	2.61
Positive	298.114	7.1	Methyl guanosine	4.37	4.05	7.81	7.87	12.65	11.63	12.87	14.50	6.95	12.89	8.11	13.54	13.84	14.32	15.45	14.64	16.14	13.20	27.97	26.46	28.09	34.06	34.89	20.39	15.40	9.10	19.53	1.54	3.29	2.01
Positive	170.092	10.8	Methyl histidine	5.83	6.00	5.63	6.43	6.03	7.07	6.73	7.18	6.16	7.73	8.95	8.96	12.39	11.64	16.87	14.15	19.43	15.85	26.48	25.09	28.76	25.83	19.21	24.77	12.25	7.02	8.81	1.80	2.09	1.80
Positive	664.114	12.0	NAD+	418.94	342.74	299.62	354.77	344.84	356.82	310.92	363.44	365.14	392.07	346.07	323.02	300.24	279.52	338.01	297.02	434.32	346.82	479.70	474.66	437.70	392.23	373.36	415.80	385.53	315.16	373.80	441.74	359.19	285.22
Positive	744.080	14.4	NADP+	26.88	24.22	22.77	23.59	24.81	20.13	22.61	22.82	25.26	25.02	25.72	22.82	20.60	17.10	23.52	19.17	25.56	24.71	22.06	22.98	23.87	19.79	17.43	17.43	15.82	24.35	22.37	7.10	9.29	5.28
Negative	184.986	14.4	Phospho-glyceric acid	312.82	202.99	188.38	229.83	294.33	171.43	208.28	259.23	178.44	287.51	253.28	302.77	106.55	112.59	121.82	139.04	113.11	147.28	209.53	253.29	246.51	275.23	206.12	222.45	155.33	181.44	101.81	0.46	0.39	0.42
Negative	166.975	15.0	Phosphoenolpyruvate	93.48	67.09	49.63	72.02	91.96	53.78	64.58	86.94	58.10	94.66	84.67	92.28	33.98	38.59	38.30	42.67	35.79	41.86	95.06	100.79	88.64	112.02	95.04	89.64	64.57	77.43	47.79	0.14	0.09	0.17
Positive	385.128	11.2	S-adenosyl-homocysteine	58.97	61.23	58.54	53.38	55.89	50.64	57.47	53.88	52.49	54.13	51.93	48.52	22.83	24.72	26.75	15.13	20.97	19.81	8.42	8.62	7.40	8.16	5.44	6.84	4.35	4.12	4.98	79.39	83.53	59.24
Positive	399.144	14.4	S-adenosyl-methionine	13.90	9.88	8.68	11.28	10.99	10.40	9.47	10.55	10.55	10.94	11.46	11.18	12.18	11.75	14.44	15.43	13.46	12.49	64.98	78.48	81.13	119.04	102.78	105.17	112.98	106.20	122.42	1.35	1.20	0.74
Negative	341.109	13.6	Trehalose	15.46	12.75	13.43	30.19	31.59	20.22	31.12	41.67	32.86	74.89	75.40	81.86	1433.44	1611.63	1570.22	2240.65	2219.84	2197.38	5399.21	5458.01	5024.08	4819.45	4467.30	4356.15	2701.37	3375.62	2766.88	4.88	2.16	2.38
Positive	198.123	10.0	Trimethyl histidine	7.32	6.99	6.55	6.77	7.46	6.98	7.09	8.50	6.77	8.55	8.94	8.32	10.58	9.11	14.61	15.15	17.61	13.41	41.49	40.83	45.04	36.85	30.36	37.69	27.36	15.76	18.84	0.70	1.13	0.89
Positive	189.159	21.1	Trimethyl lysine	2.71	2.17	2.90	2.83	2.70	1.96	2.37	2.45	2.35	2.80	2.78	3.20	3.52	2.86	3.97	3.39	4.70	4.38	8.73	9.76	10.27	10.20	8.21	8.31	6.33	16.22	7.82	0.65	1.43	0.54
Negative	606.077	12.7	UDP-acetyl-glucosamine	10.68	7.28	7.24	6.51	8.97	4.96	4.26	6.37	5.76	9.62	6.61	9.42	11.94	10.23	11.40	13.02	12.97	17.60	11.20	13.16	10.81	12.76	12.69	10.96	14.65	16.47	15.94	0.46	0.41	0.61
Negative	565.049	13.8	UDP-glucose	313.78	222.27	232.00	245.93	323.49	171.75	212.69	281.88	232.20	378.96	297.66	367.57	265.58	245.35	297.17	291.37	231.89	240.14	189.49	224.30	174.34	153.33	146.22	149.18	161.97	194.31	221.78	0.05	0.01	0.03
Negative	482.962	15.1	UTP	109.89	81.74	53.72	76.95	111.49	57.46	71.64	89.06	63.29	129.98	109.45	127.71	142.40	131.26	146.08	152.60	149.47	180.42	110.52	130.48	116.72	172.08	139.90	146.67	150.11	188.44	107.02	0.12	0.02	0.15
Negative	283.068	9.4	Xanthosine	5.35	4.71	5.23	6.12	6.47	5.05	6.14	6.30	4.65	5.84	5.75	6.07	0.94	1.10	1.15	0.70	0.65	0.73	0.74	0.62	0.80	3.37	2.88	2.61	20.06	23.65	17.90	5.08	11.06	7.26

Table S1 Metabolic compounds from cell extracts obtained from *S. pombe* cells cultured in the synthetic medium EMM2 containing 111, 22.2, 16.7, 11.1, 5.6, 4.4, 2.2, 1.7, 1.1, or 0 mM glucose for 6 h at 26°C.
For each detected compound, the normalized peak area is numerically presented with the ionization mode, m/z value, and retention time [23]. Three samples were analyzed in each condition.

Ionization mode	m/z	Retention time (min)	Compound	Normalized peak area after switch to 0 mM glucose														
				0 min #1	0 min #2	0 min #3	5 min #1	5 min #2	5 min #3	2 h #1	2 h #2	2 h #3	4 h #1	4 h #2	4 h #3	8 h #1	8 h #2	8 h #3
Positive	810.126	10.5	Acetyl-CoA	1.44	3.38	4.27	0.21	0.31	0.37	0.16	0.10	0.14	0.39	0.24	0.23	0.05	0.03	0.04
Positive	268.102	6.6	Adenosine	1.00	1.22	1.31	12.54	15.65	10.23	40.92	44.70	49.13	92.91	90.42	113.65	91.01	79.82	71.46
Negative	426.023	12.7	ADP	75.36	67.26	61.65	111.09	90.63	108.46	55.09	26.03	34.29	14.66	11.91	6.04	9.99	8.27	9.51
Negative	346.056	11.4	AMP	40.70	56.32	56.28	392.68	331.23	332.33	356.80	294.28	357.38	334.62	289.45	220.61	157.42	151.17	170.84
Negative	505.990	13.9	ATP	402.61	366.04	400.51	175.80	131.84	146.07	28.77	10.31	9.88	2.42	1.59	0.75	1.79	1.43	1.13
Positive	245.094	6.2	Biotin	11.93	13.05	16.66	13.38	17.19	8.10	0.28	0.27	0.37	0.14	0.15	0.22	0.11	0.11	0.11
Positive	489.110	13.0	CDP-choline	1.19	1.46	1.61	51.94	39.70	38.10	235.74	261.90	207.33	291.39	214.39	218.53	218.06	201.91	175.36
Positive	447.064	13.8	CDP-ethanolamine	0.32	0.40	0.38	1.81	1.77	1.30	8.66	8.76	7.65	8.30	8.00	8.66	10.12	8.99	7.41
Negative	481.979	15.6	CTP	26.76	23.35	28.24	5.43	4.32	5.61	0.40	0.10	0.09	0.00	0.00	0.00	0.00	0.00	0.00
Positive	203.149	20.7	Dimethyl arginine	3.31	4.85	4.42	4.93	4.87	2.85	4.97	4.73	5.35	3.83	4.32	5.28	4.51	4.13	4.09
Positive	312.128	10.0	Dimethyl guanosine	56.18	60.55	49.86	100.92	92.94	81.63	75.57	86.36	81.91	56.94	63.50	77.60	55.64	55.68	49.60
Positive	184.107	9.7	Dimethyl histidine	9.14	12.86	14.21	17.83	19.63	8.00	13.48	14.41	15.99	12.71	8.34	12.92	11.27	10.33	9.93
Positive	175.143	19.7	Dimethyl lysine	1.16	2.11	1.95	2.25	1.85	1.13	1.99	1.56	1.76	1.24	1.28	1.99	1.70	1.69	1.49
Positive	741.231	4.9	Ferrichrome	25.15	29.29	25.33	28.56	21.03	27.97	23.84	23.73	18.40	16.40	12.83	6.64	4.91	2.46	1.51
Negative	338.989	15.9	Fructose-1-6-diphosphate	558.28	597.13	695.08	21.65	16.86	18.43	0.27	0.04	0.05	0.01	0.02	0.00	0.00	0.00	0.00
Negative	259.023	13.4	Fructose-6-phosphate	90.17	98.17	105.04	3.73	2.04	2.17	0.37	0.12	0.15	0.06	0.05	0.03	0.04	0.04	0.02
Positive	606.080	15.4	GDP-glucose	17.53	19.71	19.12	1.07	0.80	0.82	0.10	0.09	0.11	0.08	0.07	0.05	0.03	0.05	0.05
Negative	259.023	14.4	Glucose-6-phosphate	147.30	135.02	176.10	16.59	14.31	6.23	5.24	2.39	2.48	0.47	0.45	0.25	0.29	0.31	0.26
Negative	306.077	11.9	Glutathione (GSH)	1.30	216.99	247.99	67.76	232.26	1.28	35.82	49.36	71.98	0.08	2.92	0.40	0.02	0.02	0.06
Negative	611.147	15.0	Glutathione (GSSG)	228.37	197.44	234.08	334.90	222.06	265.72	283.54	113.77	132.71	146.33	120.89	50.11	84.09	77.38	61.78
Positive	258.108	12.3	Glycerophosphocholine	936.50	1206.28	1359.65	1051.68	961.02	922.06	1292.94	1199.60	1162.21	1377.72	1070.38	1335.97	1652.03	1501.60	1277.62
Negative	214.049	13.1	Glycerophosphoethanolamine	7.81	10.11	14.81	3.60	4.10	3.55	36.94	14.01	21.88	30.73	20.88	19.18	24.07	26.47	36.00
Negative	333.059	13.6	Glycerophosphoinositol	10.06	12.67	15.15	12.66	10.09	9.74	15.40	7.89	10.14	13.93	11.07	8.80	20.00	16.99	17.77
Negative	521.985	16.4	GTP	107.05	110.05	102.80	55.52	43.37	52.33	17.33	6.98	7.73	1.45	1.19	0.29	0.23	0.26	0.10
Negative	267.073	8.4	Inosine	24.81	24.96	33.19	38.12	39.84	30.17	48.96	43.75	58.91	42.78	36.79	45.80	23.33	25.05	23.12
Positive	282.117	12.5	Methyl adenosine	6.49	5.68	6.37	13.07	11.89	8.65	9.61	9.61	9.67	6.16	3.29	8.72	4.68	4.53	3.52
Positive	298.112	7.1	Methyl guanosine	3.62	10.20	4.75	9.38	10.33	5.55	9.07	9.58	8.83	7.08	7.80	8.11	6.65	6.10	5.14
Positive	170.091	10.8	Methyl histidine	2.67	2.90	3.31	3.01	2.98	2.22	3.29	3.48	3.41	3.39	3.78	4.32	5.22	4.79	4.65
Positive	664.111	11.9	NAD+	382.44	381.85	381.68	487.52	412.15	409.79	448.58	464.05	410.08	509.21	358.07	464.64	540.06	487.90	386.50
Positive	744.076	14.4	NADP+	21.25	23.01	21.71	40.30	34.22	33.48	27.37	32.63	30.99	11.10	9.70	10.85	4.75	4.43	4.26
Negative	184.986	14.4	Phospho-glyceric acid	304.50	263.68	289.62	55.55	35.26	41.34	4.30	1.52	1.55	0.90	0.62	0.36	0.62	0.43	0.40
Negative	166.976	15.0	Phosphoenolpyruvate	64.98	54.36	66.90	13.70	9.80	11.93	0.59	0.29	0.28	0.07	0.02	0.02	0.15	0.13	0.07
Positive	385.126	11.2	S-adenosyl-homocysteine	46.92	54.35	53.62	67.87	63.70	52.72	126.57	141.33	111.06	138.61	133.61	137.73	146.99	148.52	109.14
Positive	399.142	14.4	S-adenosyl-methionine	11.42	9.63	9.76	11.82	9.11	7.98	4.55	6.25	3.73	1.56	1.54	1.48	0.95	0.93	0.60
Negative	341.109	13.6	Trehalose	9.63	8.54	10.71	5.62	4.67	4.64	1.37	0.84	0.55	1.05	0.69	0.62	1.88	1.48	1.20
Positive	198.122	10.0	Trimethyl histidine	3.81	3.68	3.81	3.78	4.24	2.23	2.36	2.80	2.82	2.06	2.10	2.16	1.94	1.93	1.67
Positive	189.158	21.1	Trimethyl lysine	1.70	2.83	2.48	2.77	2.80	1.30	2.09	2.12	2.19	1.47	1.83	1.96	1.36	1.41	1.36
Negative	606.076	12.7	UDP-acetyl-glucosamine	10.80	12.92	12.98	15.35	10.77	15.07	10.24	4.61	8.26	1.07	0.62	0.49	0.55	0.34	0.52
Negative	565.050	13.8	UDP-glucose	358.57	345.84	350.98	9.59	4.55	7.59	0.11	0.02	0.01	0.02	0.00	0.00	0.01	0.00	0.00
Negative	482.963	15.1	UTP	94.24	109.17	122.69	87.82	77.82	82.56	9.63	3.81	4.12	0.18	0.17	0.03	0.03	0.01	0.00
Negative	283.068	9.4	Xanthosine	4.74	5.19	5.08	7.06	5.83	4.42	5.35	4.75	5.64	9.17	9.45	9.95	14.13	14.65	15.07

Table S2 The time-course change in peak areas of metabolites under glucose fasting.
S. pombe cells grown at 26°C in medium containing 111 mM glucose were transferred to medium not containing glucose (fasting condition). Metabolites were extracted at the indicated times, three samples at each time point.

Ionization mode	m/z	Retention time (min)	Compound	Normalized peak area after switch to 1.1 mM glucose																				
				0 min #1	0 min #2	0 min #3	30 min #1	30 min #2	30 min #3	1 h #1	1 h #2	1 h #3	4 h #1	4 h #2	4 h #3	1 d #1	1 d #2	1 d #3	2 d #1	2 d #2	2 d #3	7 d #1	7 d #2	7 d #3
Positive	810.126	10.6	Acetyl-CoA	1.44	3.38	4.27	0.01	0.00	0.00	0.03	0.00	0.05	0.00	0.00	0.00	0.00	0.00	0.00	0.00	0.00	0.00	0.01	0.00	0.00
Positive	268.102	6.6	Adenosine	1.00	1.22	1.31	15.93	5.39	8.87	3.83	4.15	9.37	1.13	1.36	0.89	2.01	2.38	1.34	2.78	3.22	1.97	12.08	3.99	5.40
Negative	426.023	12.7	ADP	75.36	67.27	61.66	186.37	107.18	124.51	163.46	117.98	95.39	180.55	133.50	70.77	230.55	134.74	281.86	223.39	153.50	190.59	123.61	86.07	83.33
Negative	346.057	11.4	AMP	40.71	56.35	56.30	138.34	149.36	159.56	103.17	162.44	137.05	141.17	167.73	100.12	393.07	335.92	220.35	529.79	570.63	437.58	373.10	310.83	359.25
Negative	505.991	13.9	ATP	402.65	366.11	400.56	367.60	262.99	328.55	298.87	292.20	227.44	186.67	154.75	137.41	228.86	105.16	180.31	97.09	69.66	85.07	23.77	26.47	29.89
Positive	245.093	6.2	Biotin	11.93	13.05	16.66	6.31	6.62	8.82	4.83	6.40	6.16	1.19	1.71	1.18	2.48	1.77	2.52	0.14	0.09	0.12	0.01	0.00	0.00
Positive	489.109	13.0	CDP-choline	1.19	1.46	1.61	17.74	33.35	38.29	15.69	20.54	19.03	28.28	51.69	23.16	211.26	240.87	293.17	884.13	779.81	745.16	696.08	717.69	1094.92
Positive	447.063	13.8	CDP-ethanolamine	0.32	0.41	0.38	1.16	1.82	2.10	0.83	0.83	1.11	0.58	0.82	0.54	34.88	35.92	35.00	67.53	76.01	48.56	38.40	46.14	72.27
Negative	481.979	15.6	CTP	26.77	23.35	28.24	55.02	47.61	56.12	46.39	47.44	35.53	38.84	41.45	17.93	40.75	25.68	42.24	15.28	9.30	12.72	2.16	1.05	1.45
Positive	688.319	6.3	Deferriferrichrome	0.00	0.00	0.00	0.08	0.00	0.00	0.06	0.00	0.00	0.00	0.00	0.00	58.07	158.44	200.68	407.00	328.98	318.39	0.04	196.12	84.40
Positive	203.148	20.6	Dimethyl arginine	3.31	4.85	4.42	4.41	4.84	3.97	4.82	4.02	5.32	16.13	35.36	15.91	93.89	74.50	100.03	91.57	77.28	80.04	39.81	48.05	65.43
Positive	312.127	10.0	Dimethyl guanosine	55.31	59.71	49.16	3.24	54.04	37.34	21.42	55.54	42.38	58.90	56.30	50.07	47.09	37.77	54.88	16.83	15.78	15.98	4.78	2.83	3.49
Positive	184.106	9.7	Dimethyl histidine	9.13	12.86	14.20	17.02	21.02	15.05	17.78	25.96	18.59	45.17	60.74	39.59	198.94	128.39	179.31	98.74	87.93	101.75	67.02	47.35	76.84
Positive	175.142	19.7	Dimethyl lysine	1.16	2.11	1.95	2.39	1.48	1.23	1.69	1.49	1.84	5.11	9.75	4.16	25.01	22.29	32.13	22.05	19.04	21.03	8.47	8.76	10.55
Positive	230.093	12.2	Ergothioneine	0.00	0.00	0.00	0.00	0.14	0.12	0.00	0.06	0.08	2.11	3.29	1.44	41.76	33.71	50.53	29.66	42.59	41.44	19.50	23.78	39.96
Positive	741.230	4.9	Ferrichrome	25.15	29.29	25.33	35.36	21.11	35.47	46.50	33.68	33.59	97.41	69.28	79.11	654.57	265.85	398.58	548.25	521.61	473.70	598.75	373.34	279.24
Negative	338.990	15.8	Fructose-1-6-diphosphate	558.72	598.01	695.81	4.33	6.28	7.01	1.88	2.41	1.95	2.95	3.34	1.47	1.46	0.78	1.41	0.20	0.14	0.20	0.11	0.01	0.00
Negative	259.023	13.5	Fructose-6-phosphate	90.19	98.21	105.08	16.73	9.54	11.26	14.44	18.92	14.55	21.37	26.41	17.10	15.24	4.32	18.92	4.15	2.04	5.18	0.04	0.01	0.03
Negative	604.073	15.5	GDP-glucose	22.23	19.45	22.91	33.94	27.50	29.51	34.41	33.92	26.86	59.60	61.62	29.54	23.68	15.84	21.76	14.98	10.44	11.62	0.46	0.33	0.19
Negative	259.023	14.2	Glucose-6-phosphate	147.37	135.11	176.20	26.92	24.36	24.50	23.45	29.72	22.79	50.69	57.81	35.14	21.72	9.42	20.24	4.09	2.04	3.07	0.59	0.31	6.09
Negative	306.077	12.0	Glutathione (GSH)	1.29	216.15	247.36	0.04	0.49	0.55	0.08	0.47	0.16	0.05	0.02	0.01	0.00	0.00	0.00	0.02	0.04	0.00	0.11	0.00	0.00
Negative	611.148	15.0	Glutathione (GSSG)	228.39	197.46	234.10	324.85	271.83	283.49	256.25	290.84	222.89	246.51	247.69	161.48	1276.67	645.28	1056.79	1224.71	903.33	1005.19	926.82	579.37	618.12
Positive	258.108	12.3	Glycerophosphocholine	936.66	1206.48	1359.86	2933.69	2184.49	2519.92	2344.15	2235.26	2023.03	1235.79	1917.87	991.26	1662.55	965.11	1512.50	1371.50	1070.44	1248.87	887.03	725.49	1143.95
Negative	214.049	13.2	Glycerophosphoethanolamine	7.81	10.12	14.82	50.22	26.74	35.99	51.33	50.25	36.15	9.13	8.87	7.01	8.63	3.70	5.48	5.95	3.25	5.70	2.09	0.82	1.56
Negative	333.060	13.5	Glycerophosphoinositol	10.07	12.69	15.16	27.13	18.47	23.47	23.69	30.32	24.98	6.35	6.43	5.99	3.95	1.65	4.04	1.41	0.70	1.88	2.37	1.93	1.81
Negative	521.985	16.4	GTP	107.06	110.08	102.81	118.80	102.34	113.19	102.96	102.94	78.31	119.29	132.52	55.33	106.22	67.49	95.76	120.85	85.89	81.06	26.27	15.23	16.48
Negative	267.074	8.4	Inosine	24.78	24.91	33.14	52.66	67.73	69.08	78.50	119.94	87.30	103.03	89.13	92.59	30.47	21.92	28.13	0.77	1.01	1.15	6.50	4.34	2.50
Positive	282.117	12.5	Methyl adenosine	6.53	5.72	6.41	4.64	4.68	6.33	6.98	4.81	4.23	6.56	19.92	12.84	233.15	115.20	133.56	109.15	136.00	98.53	68.51	85.48	130.71
Positive	298.112	7.3	Methyl guanosine	3.64	10.24	4.77	4.00	3.23	4.47	5.64	3.83	4.11	7.79	7.03	12.40	25.55	63.05	15.21	11.66	43.33	49.97	15.31	13.20	11.89
Positive	170.091	10.9	Methyl histidine	2.67	2.90	3.30	6.18	6.56	6.11	6.98	8.34	5.85	11.34	13.81	8.32	55.77	40.64	63.16	31.65	26.70	34.85	19.44	16.63	31.49
Positive	664.110	12.0	NAD+	382.45	381.86	381.69	340.11	516.66	669.44	449.07	577.49	439.65	459.66	728.43	312.03	488.87	375.32	681.77	401.44	334.48	404.55	253.29	243.26	430.33
Positive	744.075	14.4	NADP+	21.25	23.01	21.71	11.25	23.55	30.83	27.91	19.79	16.83	25.37	34.14	18.54	17.93	20.78	33.17	17.86	15.72	11.52	10.96	11.44	21.58
Negative	184.986	14.4	Phospho-glyceric acid	304.51	263.69	289.63	88.87	67.79	80.77	77.57	90.38	69.64	126.62	146.41	81.47	100.98	44.57	92.01	13.29	11.20	11.48	1.11	1.30	1.41
Negative	166.976	15.0	Phosphoenolpyruvate	64.98	54.36	66.90	38.88	20.43	24.75	31.43	29.31	24.78	51.11	59.74	42.04	27.76	10.90	23.17	3.57	2.64	3.37	0.24	0.28	0.31
Positive	385.125	11.2	S-adenosyl-homocysteine	46.87	54.30	53.57	40.87	48.15	56.55	29.79	43.86	36.01	3.11	3.48	2.39	24.13	15.91	21.43	29.07	26.57	37.51	103.93	88.13	227.49
Positive	399.141	14.4	S-adenosyl-methionine	11.43	9.64	9.77	4.24	18.71	24.20	37.35	32.54	30.38	117.47	99.43	63.79	567.94	617.49	717.78	657.23	546.93	388.44	175.84	217.71	311.00
Negative	341.110	13.6	Trehalose	9.63	8.55	10.72	3.25	1.55	1.92	6.82	7.38	6.16	2671.36	2771.58	2866.38	6711.19	3024.31	5572.18	5452.27	4511.92	5831.50	4738.15	4100.22	4860.03
Positive	198.122	10.0	Trimethyl histidine	3.81	3.67	3.81	7.75	9.57	6.22	7.13	10.69	6.82	21.40	23.68	14.91	220.04	147.15	223.53	152.87	143.41	173.67	158.44	102.26	164.18
Positive	189.158	21.1	Trimethyl lysine	1.70	2.83	2.48	2.49	1.16	1.20	1.21	1.31	1.35	5.76	11.92	5.77	41.13	41.02	53.14	44.74	41.32	43.56	20.63	23.90	26.51
Negative	606.077	12.8	UDP-acetyl-glucosamine	10.80	12.92	12.98	50.27	19.50	24.98	27.49	12.59	8.47	37.85	30.44	21.13	29.88	20.82	39.35	26.69	16.79	21.81	12.55	13.08	13.04
Negative	565.050	13.8	UDP-glucose	358.59	345.88	351.00	272.39	224.03	290.23	343.32	308.05	236.39	527.33	401.32	368.92	297.74	198.46	265.00	119.95	83.23	112.55	1.15	1.21	0.95
Negative	482.963	15.1	UTP	94.25	109.20	122.71	69.04	63.44	78.19	54.59	48.98	36.82	72.94	76.14	39.95	118.25	65.74	133.93	46.42	37.43	37.32	26.91	16.50	24.57
Negative	283.069	9.5	Xanthosine	4.74	5.20	5.09	2.33	2.97	2.83	1.70	2.25	1.35	13.45	12.76	11.09	12.69	9.75	9.94	0.42	0.60	0.44	1.33	1.21	1.42

Table S3 The time-course change in peak areas of metabolites under glucose starvation.
S. pombe cells grown at 26°C in medium containing 111 mM glucose were transferred to 1.1-mM glucose medium for 7 days.
Metabolites were extracted at the indicated times, three samples at each time point.

Movie S1 Cells after shifting to the *fasting* condition (0 mM glucose)
The removal of glucose causes organelles standstill and eventual cell death. Significant changes in cytoplasmic features were observed after 6 h of fasting, although cell viability remained high. Cell death occurred after the arrest of gross intracellular organelles movement and the exhaustion of ATP.

Movie S2 Cells after shifting to the *starvation* condition (1.1 mM glucose)
In medium containing 1.1 mM glucose, the organelles movement was active like in cells cultured in standard EMM2 medium (111 mM glucose; see Movie S7), based on the rates of their intracellular movements, but the cells divided infrequently, suggesting that the 1/100-reduced glucose concentration of the standard medium was sufficient to sustain the organelles movement, but barely sufficient for cell division.

Movie S3 Cells after shifting to the *sub-starvation* condition (1.7 mM glucose)

Movie S4 Cells after shifting to the *severe diet* condition (2.2 mM glucose)

Movie S5 Cells after shifting to the *diet* condition (4.4 mM glucose)
When cultivated in 4.4 mM glucose, cells divided regularly with doubling time only slightly longer than that in 111 mM glucose (see text), but cell length was significantly shorter.

Movie S6 Cells after shifting to the *regular* glucose condition (11.1 mM glucose)
Cells were long and rod-shaped, and cell division was normal when cells were cultivated in 11.1 mM glucose.

Movie S7 Cells grown in culture containing *excess* (111 mM) glucose
The glucose concentration in the standard EMM2 recipe is 111 mM.
